# Heat transport across the Antarctic Slope Front controlled by cross-slope salinity gradients

**DOI:** 10.1126/sciadv.add7049

**Published:** 2023-05-03

**Authors:** Yidongfang Si, Andrew L. Stewart, Ian Eisenman

**Affiliations:** ^1^ Department of Atmospheric and Oceanic Sciences, University of California, Los Angeles, Los Angeles, CA, USA.; ^2^ Scripps Institution of Oceanography, University of California, San Diego, La Jolla, CA, USA.

## Abstract

The Antarctic Slope Front (ASF) is a strong gradient in water mass properties close to the Antarctic margins, separating warm water from the Antarctic ice sheet. Heat transport across the ASF is important to Earth’s climate, as it influences melting of ice shelves, the formation of bottom water, and thus the global meridional overturning circulation. Previous studies based on relatively low-resolution global models have reported contradictory findings regarding the impact of additional meltwater on heat transport toward the Antarctic continental shelf: It remains unclear whether meltwater enhances shoreward heat transport, leading to a positive feedback, or further isolates the continental shelf from the open ocean. In this study, heat transport across the ASF is investigated using eddy- and tide-resolving, process-oriented simulations. It is found that freshening of the fresh coastal waters leads to increased shoreward heat flux, which implies a positive feedback in a warming climate: Increased meltwater will increase shoreward heat transport, causing further melt of ice shelves.

## Teaser

High-resolution process modeling reveals a positive feedback of poleward ocean heat transport due to Antarctic ice shelf melt.

## INTRODUCTION

Recent studies have shown that the volume loss from Antarctic ice shelves is accelerating (*1*, *2*), which is largely attributed to iceberg calving (*3*–*5*) and ocean-driven basal melt (*2*, *6*). Thinning of the Antarctic ice shelf reduces buttressing, driving acceleration of mass loss from the Antarctic ice sheet (*7*–*9*) and contributing to sea level rise (*10*, *11*). Observations and model projections have indicated a widespread freshening of the Antarctic margins (*12*–*15*) possibly due to increased meltwater discharge (*15*–*17*) and changes in precipitation minus evaporation, combined with wind-driven freshwater advection by sea ice (*18*, *19*). Coastal freshening can reshape the ocean circulation around the Antarctic margins (*20*–*23*) and potentially modify the shoreward ocean heat transfer. On the other hand, additional on-shelf heat transport will lead to enhanced coastal freshening due to increased melting of ice shelves. Therefore, understanding the interplay between meltwater discharge and ocean heat transport is critical for predicting future climate change, especially for sea level rise (*24*), dense water formation, and the global overturning circulation (*25*).

**Fig. 1.**
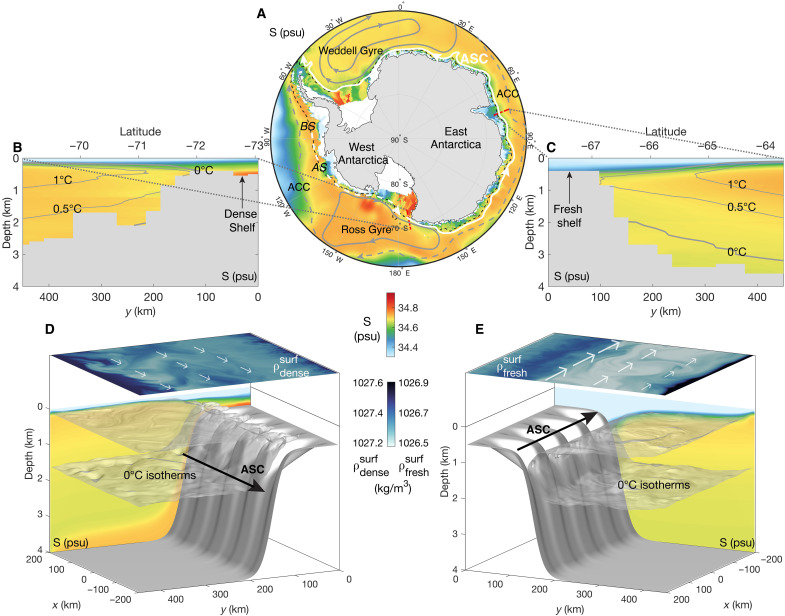
Salinity regimes around Antarctica and in our model configuration. (**A**) Climatology (1981–2010) of 500-m depth ocean salinity or seafloor salinity where the ocean is shallower than 500 m with the unit of psu (practical salinity unit). The black dashed curve indicates the 1000-m isobath. The white arrows represent the ASC, with white dashed lines in the Bellingshausen (BS), Amundsen (AS), and Ross Seas denoting the uncertain initiation of the ASC (*25*). The solid gray arrows represent the Ross Gyre and the Weddell Gyre. The dashed gray arrows represent the Antarctic Circumpolar Current (ACC). (**B**) A cross section of ocean salinity taken in the Ross Sea (73.05° to 69.01°S, 172.13°E), where the shelf is relatively salty, overlaid by gray contours of surface-referenced potential temperature. (**C**) A cross section of ocean salinity taken in East Antarctica (67.75° to 63.71°S, 76.38°E), where the shelf is relatively fresh, overlaid by gray contours of surface-referenced potential temperature. The data used in (A) to (C) come from *World Ocean Atlas 2018* (*102*). (**D**) Results of the dense-shelf (equivalent to “salty shelf” in this study) simulation with instantaneous sea surface potential density ($\rho_{\mathrm{dense}}^{\mathrm{surf}}$), 0°C isotherms, and time- and zonal-mean salinity in the background. The white arrows sketch the direction and relative strength of sea surface currents, and the black arrow schematically shows the direction of the ASC. (**E**) As (D), but shows the fresh-shelf simulation. $\rho_{\mathrm{fresh}}^{\mathrm{surf}}$ is the instantaneous sea surface potential density of the fresh-shelf simulation.

The Antarctic Slope Front (ASF) can be categorized into fresh-, dense-, and warm-shelf regimes (*25*). Each shelf regime has distinct frontal structure and dynamics. Figure 1A shows the climatology of ocean salinity at 500-m depth or at the seafloor where the ocean is shallower than 500 m. In the Ross Sea and the Weddell Sea, where the Antarctic bottom water (AABW) is formed, the coastal salinity is relatively high. Close to the Antarctic Peninsula and in East Antarctica, the continental shelves are fresher than the water masses offshore. Figure 1 (B and C) shows cross sections of ocean salinity, highlighting the “fresh shelves” and “dense shelves” around Antarctica. The arrows in Fig. 1A indicate the major ocean current systems around the Antarctic margins, with the Antarctic Slope Current (ASC) highlighted in white. As a strong gradient in water mass properties between the cold shelf water and the warmer circumpolar deep water (CDW), the ASF is essential in ocean heat transfer toward the Antarctic margins (*25*). The ASF and the associated westward ASC form a barrier to exchanges such as heat, freshwater, and nutrients between the continental shelf and the open ocean (*26*–*28*), except along the West Antarctic Peninsula (WAP) and a small sector of East Antarctica (the warm-shelf regime) (*25*). In the Bellingshausen Sea and the Amundsen Sea, where the ASC is weaker (see Fig. 1A), warm water at depth can access ice shelves via submarine troughs (*25*), leading to the highest ice shelf thinning rates around Antarctica (*1*, *2*, *29*) and thereby to coastal freshening.

Previous studies with relatively coarse resolution show that coastal freshening leads to increased shoreward heat transport, which triggers strong subsurface warming around Antarctica (*4*, *23*, *30*–*33*). For example, Golledge *et al.* (*33*) found that meltwater from Antarctica will trap warm water below the sea surface, creating a positive feedback that increases Antarctic ice loss. Some other studies have opposite predictions of ocean heat transport in response to meltwater (*34*, *35*). For example, using a global ocean-sea ice model, Moorman *et al.* (*36*) found that coastal freshening tends to isolate the continental shelves from offshore heat. However, these modeling studies did not fully resolve mesoscale eddies in their simulations due to the small Rossby radius of deformation around Antarctica (*37*, *38*), thus potentially omitted a key source of onshore ocean heat transfer contributed by eddies. Nakayama *et al.* (*39*) found increased shoreward heat transport associated with coastal freshening in East Antarctica, using a horizontal grid spacing of 3 to 4 km. However, the mechanism underlying the increased onshore heat transport remains unclear.

Though wind and buoyancy forcing have historically been recognized as key drivers of the ASC (*22*, *26*, *27*), recent studies have emphasized the role of small-scale and/or high-frequency variability in the cross-slope heat and water mass exchanges, such as mesoscale eddies (*37*, *40*–*42*), tides (*43*–*46*), dense outflows (*47*), and shelf waves (*48*, *49*). Previous studies have shown that dense AABW export enhances shoreward heat transport by eddies generated at the interface between the AABW and the CDW (*37*) or by pulses of dense shelf water outflow in canyons (*50*). For fresh shelves, eddies are shown to be important in advecting warm water into the ice shelf cavities (*22*, *40*). Using hydrographic observation and high-resolution idealized numerical simulations, Nøst *et al.* (*40*) suggested that overturning circulation induced by baroclinic eddies in the ASF brings warm deep water (WDW) onto the shelf and into the ice shelf cavity. Using hydrographic profiles of the fresh shelf at Kapp Norvegia, Hattermann (*22*) showed enhanced baroclinic eddy growth rates in summer with fresher coastal waters compared with that in winter. By incorporating eddy overturning in an idealized two-layer model, Hattermann (*22*) revealed that coastal freshening may lead to shoaling of the WDW, which allows more warm water intrusion onto the shelf. However, the tools used in these works are limited to their specific shelf regimes, and there was no comprehensive understanding of the mechanisms from a single consistent model that could be applied to different shelf regimes. Using a high-resolution, eddy- and tide-resolving regional model, Stewart *et al.* (*51*) showed the approximate cancellation between tidal and mean heat fluxes in different Antarctic sectors and emphasized the role of eddies in net onshore heat transport. They suggested that heat transport by eddy stirring along isopycnals (more precisely, “eddy diffusion”) is the principal mechanism of onshore heat transport around the Antarctic margins.

In this study, we investigate the heat transport in the fresh- and dense-shelf regimes using a single consistent model. We show that if the resolution is high enough to resolve mesoscale eddies over the continental shelf and slope, coastal freshening leads to increased shoreward heat transport. In addition, we provide insight into the dynamical mechanisms of shoreward ocean heat transport driven by eddies, tides, and mean flows, as well as its sensitivity to wind forcing, sea ice, and topography.

## RESULTS

### Shoreward heat transport in the fresh- and dense-shelf regimes

We use a high-resolution process-oriented model configuration, MITgcm_ASF (see Materials and Methods), which successfully simulates the fresh- and dense-shelf regimes of the ASF (*52*). We varied seven parameters: salinity at the southern boundary, zonal wind speed, meridional wind speed, tidal current amplitude, sea ice thickness, the width of the continental slope, and horizontal grid spacing. We choose these parameters because winds, tides, and buoyancy forcing are the key drivers of the ASC system (*25*), sea ice thickness and the geometry of the continental slope are shown to play an important role in redistributing momentum in the ASC (*52*), and the ability of numerical models to resolve mesoscale eddies strongly depends on horizontal grid spacing. The results are compared to a reference simulation that is representative of typical winter conditions of the ASF with stratified shelf water and no dense water formation (see Materials and Methods). Figure 1 (D and E) shows the instantaneous 0°C isotherms, surface potential density, and zonal-mean salinity in our simulated “dense-shelf” and “fresh-shelf” cases. The northern boundary temperature and salinity are restored to winter climatology values across the ASF (see Materials and Methods). The southern boundary temperature and salinity are set based on observations: The temperature is restored to freezing temperature throughout the water column for all the simulations, and the salinity profiles are vertically uniform for fresh shelves and increase linearly with depth for dense shelves (see Materials and Methods for details). This is equivalent to adding meltwater throughout the column near the coast. The ASC flows westward along the slope and shifts from a surface-intensified current in the fresh-shelf case (fig. S1A) to a bottom-intensified current in the dense-shelf case (fig. S1C), consistent with observations (*53*).

**Fig. 2.**
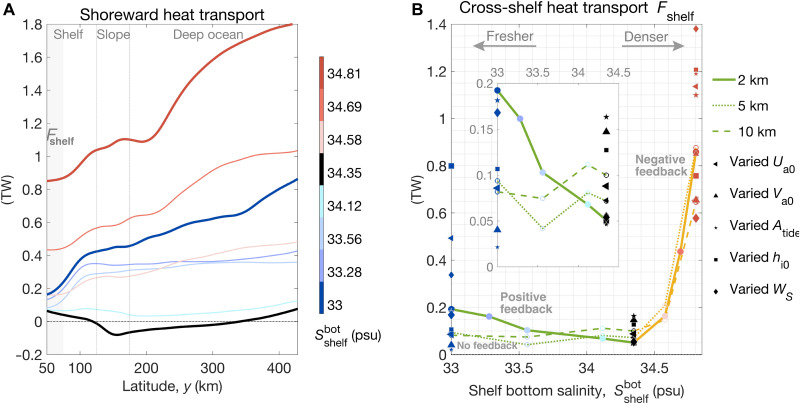
Sensitivity of shoreward heat transport to parameters varied among the simulations. (**A**) Vertically and zonally integrated meridional advective heat flux in the simulations with varied shelf bottom salinity ($S_{\mathrm{south}}^{\mathrm{bot}}$) in TW (1 TW = 10^12^ W), as a function of latitude *y*. The horizontal resolution of these simulations is 2 km. The blue, black, and red lines represent simulations with low, reference, and high shelf salinity, respectively. Light gray denotes the latitudinal band (50 to 75 km) used to calculate the averaged heat transferred onto the shelf, *F*_shelf_. The 20-km sponge layer at the northern boundary is not shown. (**B**) Heat transferred onto the continental shelf (*F*_shelf_), as a function of shelf bottom salinity prescribed at the southern boundary. The solid, dotted, and dashed lines correspond to simulations with 2-, 5-, and 10-km resolution, respectively. The green and orange curves denote the fresh- and dense-shelf regimes, respectively. Positive slopes of *F*_shelf_ in the dense-shelf regime indicate a negative feedback; negative slope of *F*_shelf_ in the fresh-shelf regime indicates a positive feedback; zero slope corresponds to no feedback. For the fresh-shelf, reference, and dense-shelf cases, the sensitivity of *F*_shelf_ to other model parameters is indicated by markers with various shapes, with larger marker sizes indicating larger values of the corresponding parameters. *U*_a0_ and *V*_a0_ are the zonal and meridional wind speed at the southern boundary, respectively. *A*_tide_ is the prescribed barotropic tidal current amplitude. *h*_i0_ is the restoring sea ice thickness at meridional boundaries, which is also the initial sea ice thickness across the domain. *W_S_* is the continental slope width. The inset plot is a zoomed-in view of *F*_shelf_ in the fresh-shelf regime.

Figure 2 shows that the sensitivity of shoreward heat transport to parameters varied among the simulations. The shoreward heat transport is largely controlled by the magnitude of the cross-slope salinity gradient (equivalently offshore buoyancy gradient), with some other parameters such as sea ice thickness and slope width greatly enhancing shoreward heat transport in the fresh-shelf and dense-shelf cases (Fig. 2B and fig. S2). The shoreward heat flux is very close to zero in the reference case, in which there is almost no offshore buoyancy gradient. Both freshening and salinification of the shelf waters relative to the offshore waters lead to increased heat flux onto the continental shelf (Fig. 2A). The heat convergence over the continental shelf is locally balanced by ocean-sea ice heat flux due to sea ice melting (fig. S3), which is sensitive to tidal amplitude. For continental shelves with large volumes of meltwater (fresh shelves), increased heat transport with freshening indicates further melt of the ice shelves, and thus, we expect a positive feedback based on the conceptual and high-resolution eddy-resolving process models of Hattermann (*22*) and previous studies (*4*, *30*, *33*). In contrast, for dense shelves, since the shoreward heat transport increases with salinification, leading to further melt and freshening, we expect a negative feedback. The cross-shelf heat flux increases more rapidly per unit shelf buoyancy change in the dense-shelf regime than in the fresh-shelf regime.

Building on the salinity gradient experiments, we covaried the following additional parameters for three groups of experiments (fresh-shelf, reference shelf salinity, and dense-shelf) (table S1): wind speeds, tidal amplitude, sea ice thickness, and continental slope width. For each group, we compared the perturbation experiments with the reference state of typical wind speeds, tides, winter sea ice thickness, and slope steepness. We additionally run simulations using coarser horizontal grid spacings (5 and 10 km) for comparison with our high-resolution (2 km) runs. To validate whether the standard eddy parameterization scheme can represent the shoreward heat transport accurately in climate models, we run these coarse simulations with and without the standard Gent-McWilliams/Redi (GM-Redi) parameterization schemes for mesoscale eddies (*54*–*56*). The perturbation experiments show that the sensitivity of shoreward heat transport to different parameters diverges for the fresh and dense shelf regimes. For fresh shelves, the shoreward heat transport increases greatly with thicker sea ice, weaker meridional or zonal winds, or steeper continental slope (fig. S2, C to H). For dense shelves, the shoreward heat transport increases with steeper continental slope, thinner sea ice, and weaker zonal wind and exhibits a nonmonotonic response to tidal amplitude (fig. S2, C to H).

**Fig. 3.**
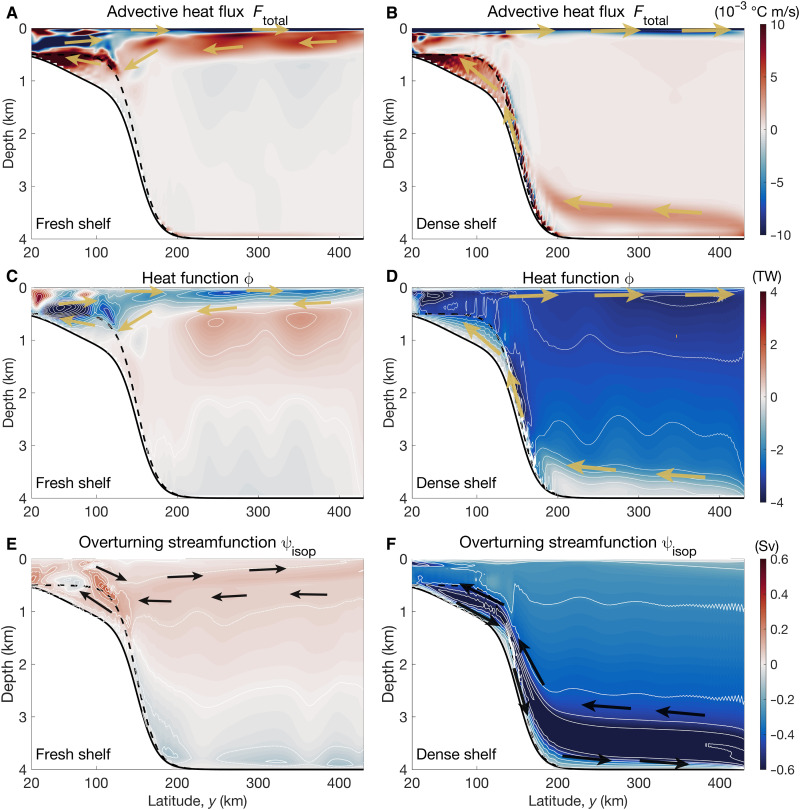
Pathways of heat and overturning circulation in the fresh-shelf and dense-shelf cases. (**A** and **B**) Time- and zonal-mean meridional advective heat flux. (**C** and **D**) Time-mean heat function in TW (1 TW = 10^12^ W). (**E** and **F**) Time-mean overturning stream function in Sv (1 Sv = 10^6^ m^3^s^−1^). Yellow arrows denote the pathways of heat, and black arrows denote the direction of the overturning circulation. (A, C, and E) The fresh-shelf case. (B, D, and F) The dense-shelf case. The spacings of the color contours in all panels are 1 of 40 of the corresponding color bar range. The black solid and dashed curves denote the deepest (bottom of the troughs) and shallowest (top of the troughs) bathymetry at each latitude (see Fig. 1, D and E), respectively. The 20-km sponge layers at the northern and southern boundaries are not shown.

The lines in Fig. 2B show the sensitivity of shoreward heat transport to horizontal grid spacing, with green and yellow lines corresponding to the fresh-shelf and dense-shelf regimes, respectively. For the cross-shelf heat transport (*F*_shelf_, shoreward heat transport averaged over the latitudinal band *y* = 50 to 75 km), the simulations with coarser resolution (5 or 10 km) are able to capture the increase of shoreward heat transport in the dense-shelf regime. Since shoreward heat transport in the dense-shelf regime is driven by dense water export through canyons (*50*) (Fig. 3, B and F), this suggests that models can capture the heat transport as long as they resolve the canyons on the shelf. However, the coarse-resolution simulations perform poorly in the fresh shelf regime because, although they can resolve deep-water eddies, they do not adequately resolve baroclinic eddies over the continental shelf (referred to as “shelf eddies” hereafter). This is because the first baroclinic Rossby radius of deformation (*57*) (close to 2.3 km over the continental shelf and 6.8 km in the deep ocean) is relatively small over the shelf. In contrast, repeating a subset of our experiments using a model resolution higher than 2 km (e.g., 1 km) does not qualitatively make any difference in reproducing the increased cross-shelf heat transport with coastal freshening in the fresh-shelf regime. It remains unclear at what horizontal resolution will the solution converge, which requires further study.

As for the cross-slope heat transport (*F*_slope_, shoreward heat transport averaged over *y* = 125 to 150 km) in the fresh-shelf regime, the 5- and 10-km simulations can capture the trend of *F*_slope_ with varied shelf bottom salinity (fig. S2B), but the magnitude of the change in *F*_slope_ is about two times smaller than that in the 2-km simulations. Note that the positive feedback in the fresh-shelf regime is related to the heat delivered onto the continental shelf (i.e., the cross-shelf heat transport) rather than the heat delivered onto the continental slope (i.e., the cross-slope heat transport). The 5- and 10-km models can partially reproduce the cross-slope heat transport, but not the cross-shelf heat transport, because the cross-slope heat transport largely depends on the tidal and mean components; since eddies are suppressed over the slope, the cross-slope heat transport does not rely on the model’s ability to resolve mesoscale eddies. In addition, the Rossby radius of deformation is larger over the slope than the shelf; thus, compared to shelf eddies, the slope eddies can be better resolved by the 5- and 10-km models. Moreover, the standard GM-Redi eddy parameterization scheme is not enough to reproduce the positive feedback, leading to even worse performance in shoreward heat transport (fig. S2, A and B), although it is possible that sophisticated “slope-aware” eddy parameterization schemes would perform better (*58*–*61*).

### Vertical structure of heat flux and overturning circulation

To provide mechanistic insight into the shoreward heat transport in different cases, we show the vertical structure of the southward advective heat fluxes in Fig. 3 (A and B) and heat function in Fig. 3 (C and D), for our high-resolution (2-km grid spacing) simulations. The heat function was first introduced by Boccaletti *et al.* (*62*) to trace the oceanic pathways of heat (see Materials and Methods for details). For both cases, the advective heat flux is offshore directly at the surface due to wind-driven Ekman transport (fig. S5, A and B). For fresh shelves, the shoreward heat transport increases in the ocean subsurface (Fig. 3, A and C). For dense shelves, the shoreward heat transport increases near the seafloor (Fig. 3, B and D).

In either case, the shoreward heat transport is associated with the overturning circulation. By vertically integrating meridional transport within potential density layers, we compute the isopycnal overturning streamfunction (*63*)

$$\psi_{\mathrm{isop}}(y,\sigma_{2})={\bar{\left\langle \int_{z=\eta_{b}}^{z=0}vH[\sigma_{2}-\sigma_{2}^{\prime }(x,y,z,t)]dz\right\rangle }}^{E}$$ (1)

where σ_2_ is the potential density with a reference depth of 2 km, σ_2_’(*x,y,z,t*) is the simulated σ_2_ field, *z* = 0 is the sea surface, *z* = η_b_ is the sea floor elevation, *v* is the meridional velocity, ℋ[⋅] is the Heaviside function, the overbar denotes a 7-year time average (Eq. 3), and the angle brackets denote zonal integral. For fresh shelves, there is a shallow overturning associated with an export of fresh surface waters (Fig. 3E) that drive warm water inflow at the subsurface. The existence of such a shallow overturning due to shelf water freshening was first proposed by Hattermann (*22*) based on observations. The deep overturning circulation in the fresh-shelf case is not connected to the continental shelf and thus does not affect the heat transport onto the shelf (Fig. 3E). For dense shelves, the overturning consists of an export of dense water near the seafloor (Fig. 3F) that drives a warmer return flow in the deep ocean.

### Role of small-scale and/or high-frequency variability

**Fig. 4.**
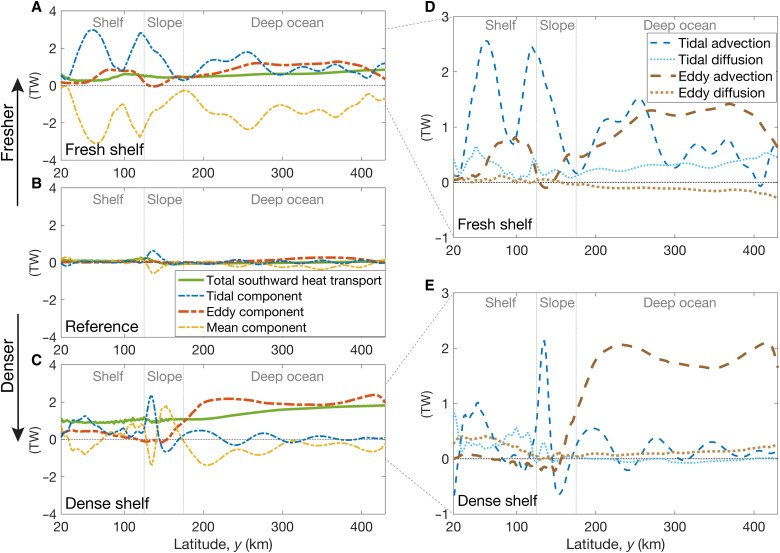
Temporal decomposition of the total shoreward heat transport. (**A** to **C**) Total shoreward heat transport, and its tidal, eddy, and mean components in the fresh-shelf, reference, and dense-shelf cases, integrated over the zonal/vertical (*x*/*z*) plane. (**D** and **E**) Tidal advection, tidal diffusion, eddy advection, and eddy diffusion in the fresh-shelf and dense-shelf cases, integrated over the zonal/vertical (*x*/σ_2_) plane. In all panels, positive values correspond to southward (shoreward) heat transport. The 20-km sponge layers at the northern and southern boundaries are not shown. Note that the tidal heat transport is largely compensated by the mean component, except for the shelf break where eddies are suppressed by a strong topographic vorticity gradient. Because of this cancellation, the tidal and mean components contribute less to the total heat transport (especially for the fresh shelves), compared with eddies.

To understand the relative contribution of tides, transient eddies, and mean flows to shoreward heat transport, we temporally decompose the total heat transport into tidal, eddy, and mean components [building on Stewart *et al.* (*51*, *64*); see Materials and Methods]. The vertical-integrated mean heat flux is negative in most regions (northward; Fig. 4, A and C) because the shoreward Ekman transport drives strong negative advective heat flux in the very surface of the ocean (fig. S5, A and B). The shoreward heat flux carried by transient eddies substantially increases over the shelf and deep ocean for both fresh and dense shelves (Fig. 4, A and C). Near the continental shelf break, the eddy heat transport is weaker (Fig. 4, A to C, and fig. S4, E and F) because the baroclinic eddies are suppressed there by strong topographic vorticity gradients (*65*–*67*). Although the tidal and mean components of the heat transport are stronger than the eddy component over the shelf and slope, their vertical integral largely compensate one another in the reference tidal regime and thus contribute less to the total heat transport (Fig. 4, A and C). For fresh shelves, the cancellation between tidal and mean components is pronounced (Fig. 4A); for dense shelves, about half of the mean component is balanced by the tidal component (Fig. 4C). The residual of this cancellation supports the heat transport across the shelf break where eddies are suppressed. Stewart *et al.* (*51*, *64*) have shown that this cancellation exists in a wide range of realistic tidal regimes around Antarctica. When tides are absent, the eddy component compensates for the mean component completely for fresh shelves (fig. S9A), leading to near-zero total onshore heat transport, while for dense shelves, there is still a large heat flux with no tides (fig. S9C and small stars in Fig. 2B and fig. S2E). Note that eddies are not the sole contributor to the increased onshore heat transport with coastal freshening, but they dominate the cross-shelf heat transport for fresh shelves and therefore needs to be carefully modeled.

We further quantify the eddy and tidal heat transport due to the net volume fluxes of water across the slope associated with advective overturning (namely, eddy advection and tidal advection) and due to the mixing of heat along isopycnals (eddy diffusion and tidal diffusion). Figure 4 (D and E) shows eddy/tidal diffusion and advection in the fresh-shelf and dense-shelf cases. For dense shelves, the eddy advection dominates the heat transport in the deep ocean, while over the continental shelf, the eddy heat transport is due to eddy diffusion (Fig. 4E). For fresh shelves, tidal advection and eddy advection are much larger than the diffusive terms (Fig. 4D), indicating that, for fresh shelves, the heat transport is due to net freshwater export. For fresh shelves, coastal freshening enhances onshore heat transfer because it increases eddy heat advection.

Inspired by the finding that eddy heat transport is essential over the shelf, we investigate the sensitivity of shelf eddies and slope eddies to the increased offshore buoyancy gradient using the energy budget. We temporally decompose the total kinetic energy into eddy, tidal, and mean components (see Materials and Methods). Although the eddies are subdominant in the total kinetic energy, the eddy kinetic energy (EKE) increases substantially when the continental shelves are very salty or very fresh, while the magnitudes of the mean kinetic energy and tidal kinetic energy do not change much across the simulations with varying shelf salinity (fig. S6, A to C). Compared to the simulation with reference shelf salinity, the zonally integrated EKE is enhanced over the shelf, upper slope, and near the surface for fresh shelves (fig. S6D), and it is enhanced over the slope and in the deep ocean for dense shelves (fig. S6F).

Since the eddy and tidal advective overturning circulations are strongly related to the advective heat transport, we also temporally decompose the isopycnal overturning streamfunction into tidal, transient-eddy, Eulerian-mean, and standing-wave components (see Materials and Methods). This is the first time that the tidal overturning streamfunction is explicitly computed. The transient-eddy component quantifies the isopycnal transport associated with transient baroclinic eddies, i.e., the deviations of the flow that have a typical time scale of the baroclinic eddies from the time-mean flow. The Eulerian-mean component quantifies the isopycnal transport associated with the time- and zonal-mean flow. The standing-wave component quantifies the isopycnal transport associated with standing eddies or standing waves, which is defined as the deviations of the time-mean flow from the time- and zonal-mean flow. It is shown that for fresh shelves, the overturning circulation is dominated by transient baroclinic eddies (fig. S7F), with the residual of mean and tidal components accounting for the overturning across the slope (fig. S7, C and E). For dense shelves, the overturning circulation is contributed by baroclinic eddies in the open ocean (fig. S8F) and a mean gravity current over the slope (fig. S8C); the bottom-intensified nature of the dense outflow makes the standing-wave component notable in the troughs of the continental shelf (fig. S8D).

With an inadequate ability to resolve small-scale variability, low-resolution simulations cannot accurately reproduce eddy and tidal heat advection (figs. S10 and S11) and thus cannot capture the positive feedback in the fresh-shelf regime. For the fresh-shelf regime, tidal advection decreases with coarser horizontal resolution, while the eddy advection exhibits a nonmonotonic response to resolution (figs. S10D and S11D), which may be due to the spurious mixing in the low-resolution model and the resulting enhancement of eddy overturning over the continental shelf (figs. S16F and S17F). Stewart (*68*) has reported a similar enhancement of eddy overturning circulation with decreased model resolution in a regional model of the Weddell Sea, albeit in a dense shelf regime.

## DISCUSSION

**Fig. 5.**
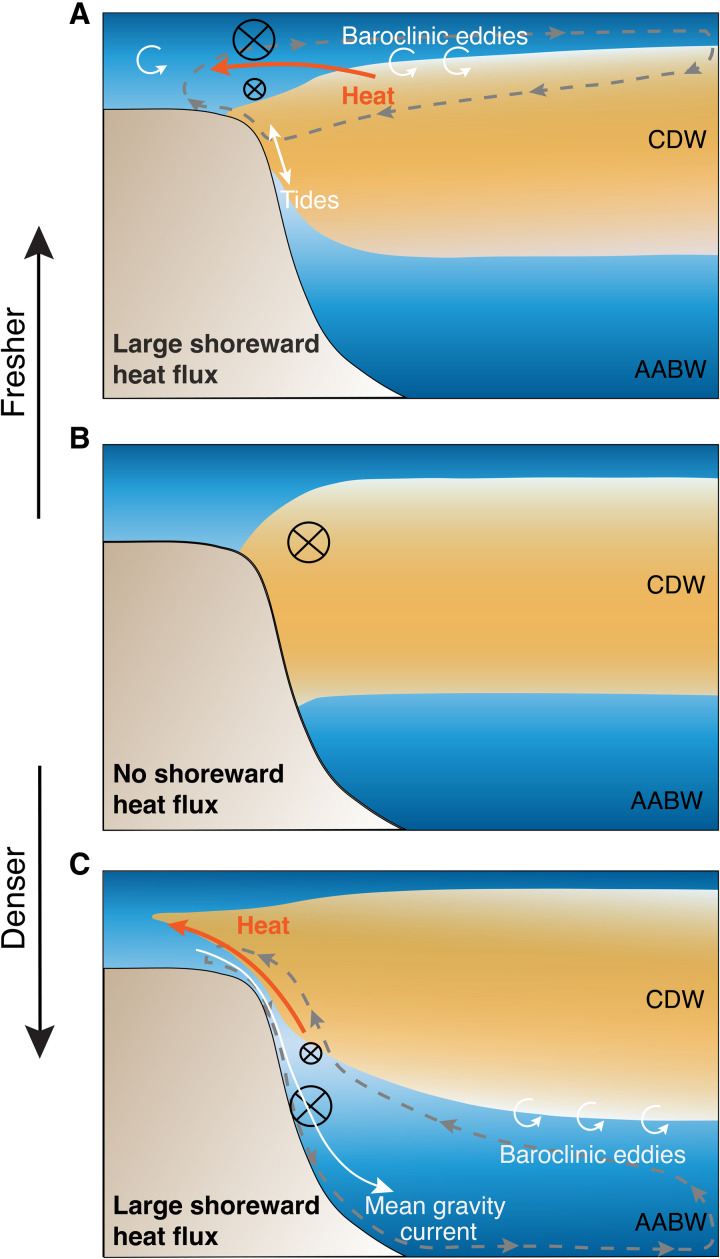
Schematic of salinity-controlled shoreward heat transport. (**A**) Fresh-shelf case. (**B**) Reference case. (**C**) Dense-shelf case. The gray dashed curves with arrows denote the zonally averaged meridional overturning circulation. The black circle with a cross shows that the direction of the slope current is westward (into the page), with the size of the circle representing the strength of the slope current. The circular arrows denote the transient baroclinic eddies. In (C), the white curve with an arrow denotes the mean gravity current associated with dense water outflow.

In conclusion, both freshening of the fresh shelves and salinification of the dense shelves lead to enhanced heat flux toward the Antarctic margins. Figure 5 schematically summarizes the processes responsible for enhanced shoreward heat transport for fresh shelves and dense shelves. Freshening the shelf leads to baroclinic instabilities of the slope front that drive a shallow overturning, bringing warm waters onto the shelf and exporting fresh surface waters offshore (Fig. 5A), while salinifying the shelf leads to dense outflows in canyons that drive a warm return flow at mid-depth (Fig. 5C). For fresh shelves, tides are important to transfer heat across the shelf break where eddies are strongly suppressed. The increased onshore heat transfer with coastal freshening in the fresh-shelf regime happens only when the horizontal resolution is high enough, and thus the model is capable of resolving mesoscale eddies over the shelf and slope (Fig. 2). These mechanisms of shoreward mass/heat transfer are consistent with the findings of Stewart and Thompson (*37*) and Morrison *et al.* (*50*) for dense shelves, Nøst *et al.* (*40*) and Hattermann (*22*) for fresh shelves, and previous studies on buoyancy-driven coastal currents in other parts of the ocean (*69*, *70*). Consistent with Stewart *et al.* (*51*), in our model, there is a notable cancellation between mean heat transport and tidal heat transport over the shelf and deep ocean; in contrast to their findings, we show that it is the eddy advection rather than eddy diffusion that dominates the cross-shelf heat transport in the fresh-shelf regime.

Our results imply a positive feedback for future climate change: In a warming climate, large volumes of meltwater may increase shoreward heat transport, causing further melt of ice shelves. The enhanced heat transport with weaker zonal winds in the fresh-shelf and dense shelf cases further implies an increased shoreward heat transport in the future, as weakening easterlies may be expected in the future due to warming over Antarctica and southward shift of the westerlies (*71*). Our results imply that the potential tipping point of the Filchner-Ronne Ice shelf may be affected not only by cavity circulation [e.g., Hellmer *et al.* (*20*)] but also by the response of dense shelves to reduced dense water export in the central and western Weddell Sea, as well as the response of fresh shelves to increased meltwater export in the eastern Weddell Sea. Furthermore, our results imply that models with coarse horizontal resolution tend not to capture the enhanced shoreward heat flux under Antarctic margin freshening, because they do not resolve shelf eddies and therefore cannot accurately represent the response of eddy advection to the increased density gradient across the ASF in the fresh-shelf regime. Therefore, coarse-resolution climate model projections of future Antarctic ice shelf melt and Antarctic coastal ocean changes should be interpreted with caution. For future research on ocean heat transport and ice shelf melting, it is essential to use high-resolution eddy-resolving models and/or improve parameterization schemes for eddy heat flux over the continental shelf and slope. Note that other physical mechanisms may contribute to the positive or negative feedback simultaneously, such as trapping of warm water in the ocean subsurface due to increased shelf stratification under coastal freshening [e.g., Golledge *et al.* (*33*)] and the enhancement of shelf circulation due to additional meltwater fluxes in the warm-shelf regime (*21*), which cannot be ruled out by the findings of this study.

There are some caveats due to the heavy idealization of the model (see Materials and Methods). In particular, models with periodic zonal boundaries may overemphasize eddy- and tidal-driven onshore transport because, in reality, meltwater can flow away along the coast without crossing the shelf break by eddy-driven and tidally driven overturning circulation. Moreover, our conclusions cannot be directly applied to the warm-shelf regime. Although it carries various caveats, the eddy- and tidal-resolving process model successfully reproduces the salient features of the fresh and dense shelves around Antarctica (*52*). It helps us disentangle the contributions from eddies, tides, standing waves, and Eulerian mean flows and allows us to explore wide parameter regimes. The idealized model confirms an important climate feedback proposed by Hattermann (*22*) that may be key to projecting future changes of Antarctic ice sheets and thus sea level rise. The results of this study help to refine predictions of future changes in different salinity regimes of the Antarctic margins and have implications on eddy heat transport across fronts in other parts of the ocean.

## MATERIALS AND METHODS

### Model configuration and simulations

In this study, we use a high-resolution process-oriented model (MITgcm_ASF) developed by Si *et al.* (*52*), based on the Massachusetts Institute of Technology General Circulation Model (MITgcm) (*72*, *73*). We use an ocean/sea ice model because the ocean-sea ice interaction exerts an important dynamical influence on the ASF/ASC (*25*, *52*). The model solves the hydrostatic Boussinesq equations with a high-order polynomial approximation to the equation of state (*74*). The model simulates sea ice dynamics (*75*) using a viscous plastic ice rheology (*76*) and thermodynamics with seven sea ice thickness categories (*77*). We need to use high resolution to resolve mesoscale eddies over the shelf and slope (*38*, *42*), and hence, we adopt a small domain (450 km by 400 km by 4 km) and fine horizontal grid spacing (2 km). The vertical grid spacing ranges from 10 m at the surface to 100 m at the bottom with 70 vertical levels. We add troughs to the model bathymetry, with the depths and widths of the troughs selected on the basis of observations (*78*), to (i) better represent the geometry of the Antarctic continental shelf and slope, (ii) allow topographic form stress to remove momentum at the seafloor (*52*, *79*), and (iii) allow dense water export through these submarine canyons (*50*). Note that we run highly idealized simulations and do not seek to accurately recreate the ocean geometry or synoptic forcing.

The model represents typical annual-mean conditions of the fresh- and dense-shelf regimes of the ASF. We exclude seasonal variations from our simulations and set the atmospheric properties to minimize the net air-ice thermodynamic fluxes and thus preserve a relatively uniform sea ice cover, because this is the representative of the typical conditions in Antarctica (*52*). Idealized wind forcing and tidal currents are imposed based on typical conditions observed near the Antarctic margins (*80*–*84*). The model has periodic zonal boundaries and open boundary conditions for the meridional boundaries that enable the implementation of tidal currents. We impose the bulk meridional density gradient as a control parameter via two 20-km-wide sponge layers at the northern and southern boundaries. The relaxation time scales are 10 and 0.5 days for the inner and outer boundaries of the sponge layers, respectively.

At the northern boundary, we restore the ocean temperature and salinity to the winter climatology of hydrography across the ASF, taken at Kapp Norvegia (71°S, 17°W) (*85*). At the southern boundary, the potential temperature is vertically uniform and equal to the freezing temperature, as typically observed (*86*), and we impose a linear vertical profile of salinity. We control the offshore density gradient by varying the maximum salinity and salinity gradient at the southern boundary (table S1):

1) For the reference simulation, the surface salinity of the southern boundary is set to be the same as the surface salinity of the northern boundary [34.12 practical salinity units (psu)]; the bottom salinity of the southern boundary (shelf bottom salinity) is selected to make sure that there is no dense water formation over the shelf; i.e., the potential density with a reference pressure of 4 km (σ_4_) at the bottom of the continental shelf (*y* = 0 km, *z* = −500 m) is identical to that at the seafloor of the northern boundary (*y* = 450 km, *z* = −4000 m).

2) For simulations with shelf salinity fresher than the reference case (“fresh-shelf regime”), we use a vertically uniform salinity profile at the southern boundary.

3) For simulations with shelf salinity saltier than the reference case (“dense-shelf regime”), we set the surface salinity to be 34.12 psu and then increase salinity linearly with depth. Under our model configuration, the shelf bottom salinity (or, equivalently, the maximum meridional salinity gradient at mid-depth) is the key, while the vertical gradient of salinity at the southern boundary makes a smaller impact.

We set the restoring salinity in this way according to the climatology (Fig. 1, B and C), i.e., large vertical variation of shelf salinity in the dense-shelf regime and much smaller vertical variation of shelf salinity in the fresh-shelf regime. For the dense shelf, the average and maximum squared buoyancy frequency *N*^2^ are 1.7 × 10^−6^ and 7.4 × 10^−5^ s^−2^ (2.1 × 10^−6^ and 4.4 × 10^−5^ s^−2^) in the steady state of the simulation (the cross section of WOA 2018), respectively. For the fresh shelf, the average and maximum *N*^2^ are 2.7 × 10^−6^ and 1.1 × 10^−4^ s^−2^ (2.1 × 10^−6^ and 1.4 × 10^−4^ s^−2^) in the simulation (the cross-section of WOA 2018), respectively. In all cases, the change of the Rossby radius of deformation is dominated by the change of bathymetry: The first baroclinic Rossby radius of deformation is about 2 to 3 km over the shelf, 3 to 10 km over the slope, and ~8 km in the deep ocean.

The model is forced by a fixed atmospheric state. Both the zonal and meridional winds are strongest at the southern boundary (*y* = 0 km), decreasing linearly offshore with zero wind speeds at the northern boundary (*y* = 450 km). In addition, we prescribe a barotropic tidal current in the meridional direction normal to the northern and southern boundaries, with an idealized tidal period of 12 hours. Because of mass conservation, the tidal current amplitude over the shelf is about eight times larger than that in the deep ocean (*87*). For a detailed description of model bathymetry, atmospheric state, tides, sea ice, initial and boundary conditions, viscosity, and diffusivity, see Si *et al.* (*52*).

In contrast to Si *et al.* (*52*), we use the Smagorinsky viscosity (*88*, *89*) in all the simulations of this study. The Smagorinsky viscosity is based on Kolmogorov’s theory of 3D homogeneous turbulence (*90*) and is effective in maintaining numerical stability without suppressing the energy of resolved scales (*91*). We turn off the grid-dependent biharmonic viscosities and set the nondimensional Smagorinsky biharmonic viscosity factor to 4. In addition, we apply standard GM-Redi eddy parameterization (*54*–*56*) for simulations with a horizontal grid spacing of 5 or 10 km. The isopycnal diffusivity and thickness diffusivity are set to 100 m^2^/s. The maximum isopycnal slope is 0.025. The Danabasoglu–McWilliams (1995) (DM95) tapering scheme (*92*) is activated for these simulations, with a DM95 critical slope of 0.025 and a DM95 tapering width of 0.0025. The gray lines in fig. S2 (A and B) show that the straightforward application of the GM-Redi parameterization cannot correctly capture the onshore heat transport. We experimented with additional combinations of GM-Redi parameters, not reported here, but were unable to obtain an improved representation of the onshore heat transport.

Table S1 shows the list of experiments. Seven model parameters are varied: (i) shelf salinity profile, including the restoring salinity at the sea surface ($S_{\mathrm{south}}^{\mathrm{surf}}$) and the seafloor ($S_{\mathrm{south}}^{\mathrm{bot}}$) of the continental shelf at the southern boundary; (ii) zonal wind speed at the southern boundary (*U*_a0_); (iii) meridional wind speed at the southern boundary (*V*_a0_); (iv) barotropic tidal current amplitude (*A*_tide_) at the northern boundary; (v) restoring sea ice thickness (*h*_i0_) at the southern boundary; (vi) continental slope width (*W_S_*); and (vii) horizontal grid spacing (Δ*_x_*, Δ*_y_*). Each simulation is run for 20 years to spin up from a stationary state, followed by a 7-year analysis period. All the results shown in this manuscript are based on data from the 7-year analysis period.

Figure S1 shows the time- and zonal-mean zonal circulation, potential temperature, and salinity in the fresh-shelf, reference, and dense-shelf cases, overlaid by neutral density contours. As reported by previous studies (*52*, *53*), the slope current is surface-intensified in the fresh-shelf case (fig. S1A), nearly barotropic in the reference case (fig. S1B), and bottom-intensified in the dense-shelf case (fig. S1C). Undercurrents appear for large salinity gradients, flowing eastward in the fresh-shelf and dense-shelf cases (fig. S1, A and C).

### Model limitations

There are several limitations of this work due to the heavy idealization of the model, which was required to allow adequate resolution of eddies.

1) The model simulates typical annual-mean conditions of the Antarctic margins with permanent sea ice cover, with the assumption that most of the freezing happens south of the model domain and most of the melt happens to the north. It does not include atmospheric feedbacks and seasonal melting/freezing of sea ice, which strongly influence ocean stratification, and thus may modulate shoreward ocean heat transport (*22*, *93*).

2) The model does not simulate the dynamics and thermodynamics of ice shelves and thus also lacks ice shelf-ocean interactions.

3) When meltwater is injected under ice shelves, it increases both the vertical stratification and the lateral density gradient. Ice shelf meltwater in previous global-scale models is usually injected only on the ocean surface (*23*, *32*, *36*), while observations and idealized modeling studies have suggested that the injection of meltwater occurs at depth (*94*–*96*). In this study, we focus primarily on the change of lateral density gradient in response to additional meltwater. We did not investigate how ocean heat transport responds to the depth at which meltwater is injected and defer this question to a future study.

4) There is a limitation associated with the re-entrant channel: The periodic boundaries efficiently remove zonal pressure gradients and meltwater/heat advection from upstream; thus, the model may overemphasize eddy- and tidal-driven transport. In reality, meltwater can flow away to the west as a coastal current, without crossing the shelf break by eddy-driven and tidally driven overturning circulation, and, as a result, the feedback may occur differently. A high-resolution circumpolar regional model is needed to understand the effect of meltwater advection along the coast (*97*, *98*).

5) The parameter regime spanned by the model experiments is not representative of the warm-shelf regime along the WAP, where the ice shelves are melting rapidly (*1*, *2*), because the model’s shelf forcing imposes a strong temperature gradient at the southern boundary that differs from conditions along the WAP. Therefore, our results cannot be directly applied to the warm-shelf regime, where previous studies such as Nakayama *et al.* (*99*) highlight heat transport carried by mean ocean currents. An idealized model that represents the warm-shelf regime with coupled ocean, sea ice, and ice shelves is needed to better understand the mechanisms and feedbacks.

6) The model bathymetry is also highly idealized. The width and depth of the troughs are selected on the basis of the typical values in observations (*78*), but we did not test the sensitivity of heat transport to the geometry of the troughs.

### Heat function

The heat function (*62*) is defined as

$$\phi (y,z)=c_{p}\rho_{0}\;\int_{z^{\prime }=\eta_{b}}^{z}\langle {\bar{v\theta }}^{E}-{\bar{v}}^{E}\theta_{\mathrm{ref}}\rangle dz^{\prime }$$ (2)

where *c_p_* is the specific heat capacity, ρ_0_ is the reference density, η_b_ is the seafloor elevation, *v* is the meridional velocity, θ is the potential temperature, θ_ref_ is the reference potential temperature, ${\bar{\cdot }}^{E}$denotes a 7-year time average, and the angle brackets denote zonal integral. The interpretation of the heat function is very similar for different choices of the reference temperature (*62*), so we use θ_ref_ = 0°C in this study for simplicity.

### Operators for temporal decomposition

To calculate temporal decomposition of shoreward heat transport, overturning streamfunction, and kinetic energy, we define two time averages over a single day (${\bar{\cdot }}^{T}$) and over the analysis period (${\bar{\cdot }}^{E}$) (*51*, *64*)

$${\bar{\cdot }}^{E}=\frac{1}{1\;\mathrm{day}}\int_{t_{0}}^{t_{0}+1\;\mathrm{day}}\cdot dt,{\bar{\cdot }}^{E}=\frac{1}{7\;\mathrm{years}}\int_{t_{0}}^{t_{0}+7\;\mathrm{years}}{\bar{\cdot }}^{T}dt$$ (3)

These operators allow us to decompose any simulation variable into mean (ξ*_m_*), eddy (ξ*_e_*), and tidal (ξ*_t_*) components

$$\xi_{m}={\bar{{\bar{\xi }}^{T}}}^{E}={\bar{\xi }}^{E},\xi_{e}={\bar{\xi }}^{T}-{\bar{\xi }}^{E},\xi_{t}=\xi -\xi_{m}-\xi_{e}=\xi -{\bar{\xi }}^{T}$$ (4)

where ξ represents velocity ***u*** = (*u, v, w*), potential temperature θ, or potential density σ_2_ with a reference depth of 2 km.

### Decomposition of the total meridional heat transport

The total meridional heat transport in the ocean is almost entirely contributed by the advective heat transport. Following Stewart *et al.* (*51*), we temporally decompose the total southward advective heat flux (*F*_total_) into mean (*F*_mean_), eddy (*F*_eddy_), and tidal (*F*_tide_) components, i.e., *F*_total_ *= F*_mean_ *+ F*_eddy_ *+ F*_tide_.

$$F_{\mathrm{total}}=-{\bar{v\theta }}^{E}$$ (5)

$$F_{\mathrm{mean}}=-v_{m}\theta_{m}$$ (6)

$$F_{\mathrm{eddy}}=-{\bar{v_{e}\theta_{e}}}^{E}=-{\bar{{\bar{v}}^{T}\;{\bar{\theta }}^{T}}}^{E}-F_{\mathrm{mean}}$$ (7)

$$F_{\mathrm{tide}}=-{\bar{v_{t}\theta_{t}}}^{E}=F_{\mathrm{total}}+{\bar{{\bar{v}}^{T}\;{\bar{\theta }}^{T}}}^{E}$$ (8)

Figure 4 (A to C) shows the zonally and vertically integrated southward advective heat fluxes. In all cases, the southward tidal heat transport is largely compensated by the offshore mean component, which is consistent with Stewart *et al.* (*51*). The residual of the mean and tidal components comprises the heat transport near the shelf break. Relative to the reference case, the eddy heat transport is enhanced over the shelf and in the deep ocean in both the fresh-shelf and dense-shelf cases (Fig. 4, A to C, and fig. S4, E and F).

### Diffusion and advection by tides and eddies

We further decompose the isopycnal thickness flux (*T*_total_, integrated meridional transport in each isopycnal layer) into mean (*T*_mean_), eddy (*T*_eddy_), and tidal (*T*_tide_) components, i.e., *T*_total_ *= T*_mean_ *+ T*_eddy_ *+ T*_tide_.

$$T_{\mathrm{total}}(x,y,\sigma_{2})={\bar{vh^{\mathrm{isop}}}}^{E}$$ (9)

$$T_{\mathrm{mean}}(x,y,\sigma_{2})=v_{m}h_{m}^{\mathrm{isop}}$$ (10)

$$T_{\mathrm{eddy}}(x,y,\sigma_{2})={\bar{v_{e}h_{e}^{\mathrm{isop}}}}^{E}={\bar{{\bar{v}}^{T}\;{\bar{h^{\mathrm{isop}}}}^{T}}}^{E}-T_{\mathrm{mean}}$$ (11)

$$T_{\mathrm{tide}}(x,y,\sigma_{2})={\bar{v_{t}h_{t}^{\mathrm{isop}}}}^{E}=T_{\mathrm{total}}-{\bar{{\bar{v}}^{T}\;{\bar{h^{\mathrm{isop}}}}^{T}}}^{E}$$ (12)

Here, *h*^isop^(*x*, *y*, σ_2_, *t*) is the isopycnal layer thickness. We remap the time-mean potential temperature to the potential density coordinates, ${\bar{\hat{\theta }(x,y,\sigma_{2})}}^{E}$, and then calculate tidal advection, tidal diffusion, eddy advection, and eddy diffusion, following Stewart and Thompson (*37*):

$$F_{\mathrm{eddy}}^{\mathrm{adv}}=-T_{\mathrm{eddy}}{\bar{\hat{\theta }}}^{E},F_{\mathrm{tide}}^{\mathrm{adv}}=-T_{\mathrm{tide}}{\bar{\hat{\theta }}}^{E}$$ (13)

$$F_{\mathrm{eddy}}^{\mathrm{diffusion}}=F_{\mathrm{eddy}}-F_{\mathrm{eddy}}^{\mathrm{adv}},F_{\mathrm{tide}}^{\mathrm{diffusion}}=F_{\mathrm{tide}}-F_{\mathrm{tide}}^{\mathrm{adv}}$$ (14)

Eddy advection and tidal advection quantify the cross-slope heat transport associated with the net volume fluxes. The eddy and tidal diffusion quantify the mixing of heat along isopycnals (*37*). Positive values correspond to southward heat transport.

### Decomposition of the total kinetic energy

The total kinetic energy (KE) is decomposed into mean (MKE), eddy (EKE), and tidal (TKE) components, i.e., KE = MKE + EKE + TKE.

$$\mathrm{KE}=\frac{1}{2}{\bar{u^{2}}}^{E}$$ (15)

$$\mathrm{MKE}=\frac{1}{2}{\bar{u_{m}^{2}}}^{E}$$ (16)

$$\mathrm{EKE}=\frac{1}{2}{\bar{u_{e}^{2}}}^{E}=\frac{1}{2}{\bar{{\left({\bar{u}}^{T}\right)}^{2}}}^{E}-\mathrm{MKE}$$ (17)

$$\mathrm{TKE}=\frac{1}{2}{\bar{u_{t}^{2}}}^{E}=\mathrm{KE}-\frac{1}{2}{\bar{{\left({\bar{u}}^{T}\right)}^{2}}}^{E}$$ (18)

### Decomposition of the isopycnal overturning streamfunction

As noted in the section "Vertical structure of heat flux and overturning circulation", the shoreward heat transport is closely related to the meridional overturning circulation. We investigate to what extent different components of the flow contribute to the overturning circulation by decomposing the isopycnal overturning streamfunction (ψ_isop_) into mean (ψ_mean_), transient-eddy (ψ_eddy_), and tidal (ψ_tide_) components, i.e., ψ_isop_ = ψ_mean_ + ψ_eddy_ + ψ_tide_. We calculate the overturning streamfunction in potential density (σ_2_) coordinates and then remap it to *z* coordinate following the standard approach (*100*)

$$\psi_{\mathrm{isop}}(y,\sigma_{2})={\bar{\left\langle \int_{z=\eta_{b}}^{z=0}vH[\sigma_{2}-\sigma_{2}^{\prime }(x,y,z,t)]dz\right\rangle }}^{E}$$ (19)

$$\psi_{\mathrm{mean}}(y,\sigma_{2})=\left\langle \int_{z=\eta_{b}}^{z=0}v_{m}H[\sigma_{2}-\sigma_{2m}^{\prime }(x,y,z)]dz\right\rangle$$ (20)

$$\psi_{\mathrm{eddy}}(y,\sigma_{2})={\bar{\left\langle \int_{z=\gamma_{b}}^{z=0}v_{e}H[\sigma_{2}-\sigma_{2e}^{\prime }(x,y,z,t)]dz\right\rangle }}^{E}$$ (21)

$$={\bar{\left\langle \int_{z=\eta_{b}}^{z=0}{\bar{v}}^{T}H[\sigma_{2}-{\bar{\sigma_{2}^{\prime }(x,y,z,t)}}^{T}]dz\right\rangle }}^{E}-\psi_{\mathrm{mean}}(y,\sigma_{2})$$

$$\psi_{\mathrm{tide}}(y,\sigma_{2})={\bar{\left\langle \int_{z=\eta_{b}}^{z=0}v_{t}H[\sigma_{2}-\sigma_{2t}^{\prime }(x,y,z,t)]dz\right\rangle }}^{E}$$

$$=\psi_{\mathrm{isop}}(y,\sigma_{2})-{\bar{\left\langle \int_{z=\eta_{b}}^{z=0}{\bar{v}}^{T}H[\sigma_{2}-{\bar{\sigma_{2}^{\prime }(x,y,z,t)}}^{T}]dz\right\rangle }}^{E}$$ (22)

Here, primes (′) denote simulated field, ℋ[⋅] is the Heaviside function, and the angle brackets denote the zonal integral. We further decompose the mean overturning streamfunction into Eulerian-mean (ψ_EM_) and standing-wave (ψ_SW_, also referred to as “standing-eddy”) components, i.e., ψ_mean_ = ψ_EM_ + ψ_SW_.

$$\psi_{\mathrm{EM}}(y,\sigma_{2})=\int_{z=\eta_{b}}^{z=0}\langle v_{m}\rangle H[\sigma_{2}-\langle \sigma_{2m}(x,y,z)\rangle ]dz$$ (23)

$$\psi_{\mathrm{SW}}(y,\sigma_{2})=\psi_{\mathrm{mean}}(y,\sigma_{2})-\psi_{\mathrm{EM}}(y,\sigma_{2})$$ (24)

Figures S7 and S8 show the temporal decomposition of the isopycnal overturning streamfunction for fresh and dense shelves with 2-km resolution. For fresh shelves, there is a subsurface, baroclinic, eddy-driven overturning over the continental shelf and in the open ocean (fig. S7F); the tidal overturning is approximately compensated by the Eulerian-mean overturning, with the residual supporting the overturning across the continental slope (fig. S7, C and E). For dense shelves, the transient eddies dominate the overturning in the deep ocean (fig. S8F); gravity currents comprise the Eulerian-mean component over the slope (fig. S8C) where eddies are suppressed; the standing eddy component dominates the isopycnal transport in the troughs on the continental shelf (fig. S8D).
