# Supplementary material for: Heat transport across the Antarctic Slope Front controlled by cross-slope salinity gradients
Source: Sci Adv. 2023 May 3;9(18):eadd7049. doi: 10.1126/sciadv.add7049 (PMC10156111; doi:10.1126/sciadv.add7049)
Supplement: Supplementary file 1 — Figs. S1 to S21 Table S1 [file sciadv.add7049_sm.pdf]

Science Advances
AAAS

Supplementary Materials for

Heat transport across the Antarctic Slope Front controlled by cross-slope salinity gradients

Yidongfang Si *et al.*

Corresponding author: Yidongfang Si, ysi@g.ucla.edu

Sci. Adv. **9**, eadd7049 (2023)
DOI: 10.1126/sciadv.add7049

This PDF file includes:

Figs. S1 to S21
Table S1

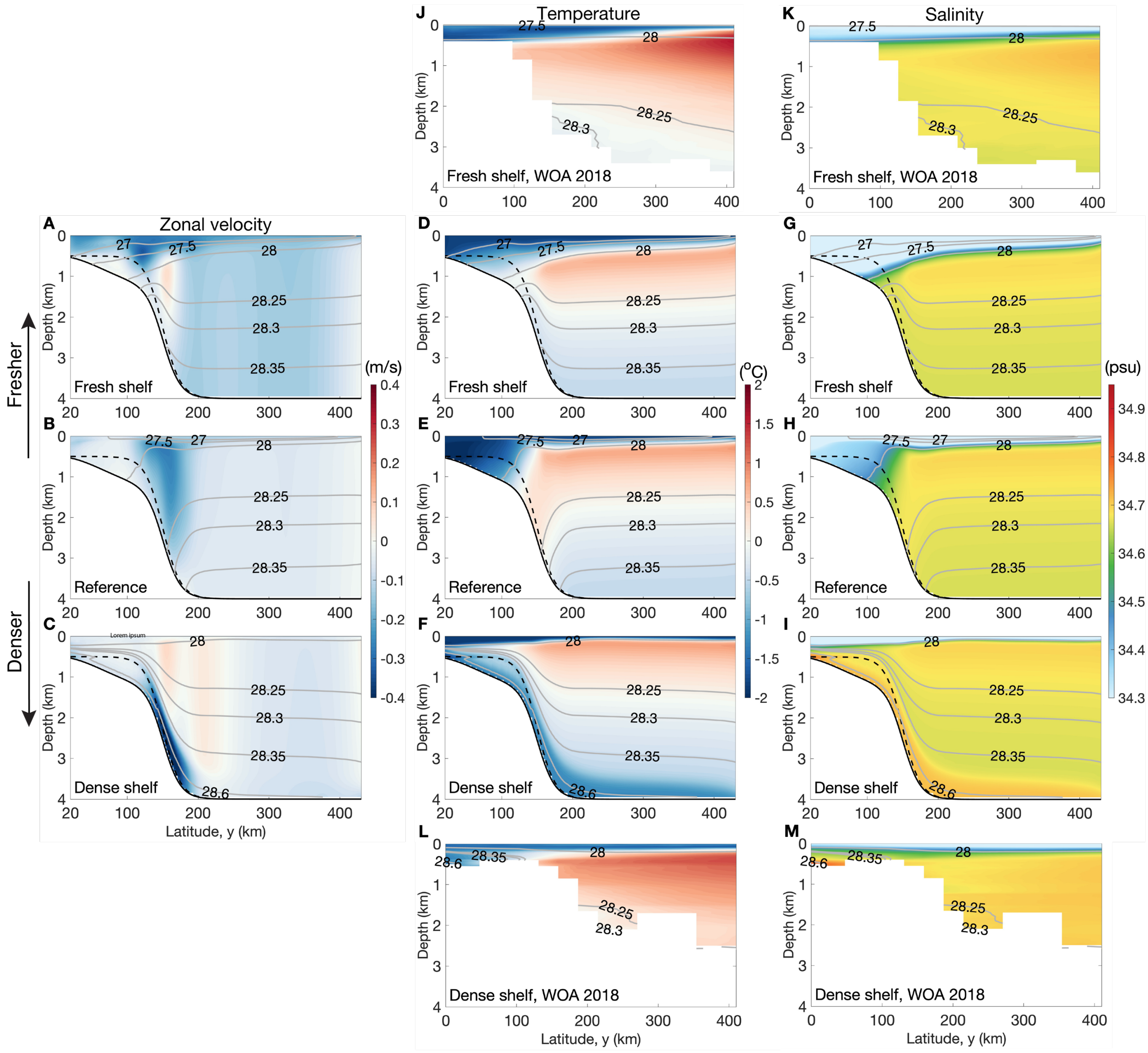

Fig. S1. Time- and zonal-mean zonal (along-slope) velocity (A-C), potential temperature (D-F), and salinity (G-I) of the simulations (2-km resolution), overlaid by neutral density contours. (**A**, **D**, **G**) The fresh-shelf case. (**B**, **E**, **H**) The reference case. (**C**, **F**, **I**) the dense-shelf case. In panels (**A-C**), blue denotes westward flow (into the page), and red denotes eastward flow (out of the page). The gray contours with numbers show the time- and zonal-mean neutral density (kg/m^3). The black solid and dashed curves denote the deepest and shallowest bathymetry (see Figs. 1D, E), respectively. The 20-km sponge layers at the northern and southern boundaries are not shown. (**J-M**) Cross sections of potential temperature and salinity taken in East Antarctica (fresh shelf, 67.75°-63.71°S, 76.38°E) and the Ross Sea (dense shelf, 73.05°-69.01°S, 172.13°E), respectively. Data comes from World Ocean Atlas 2018 (*42*).

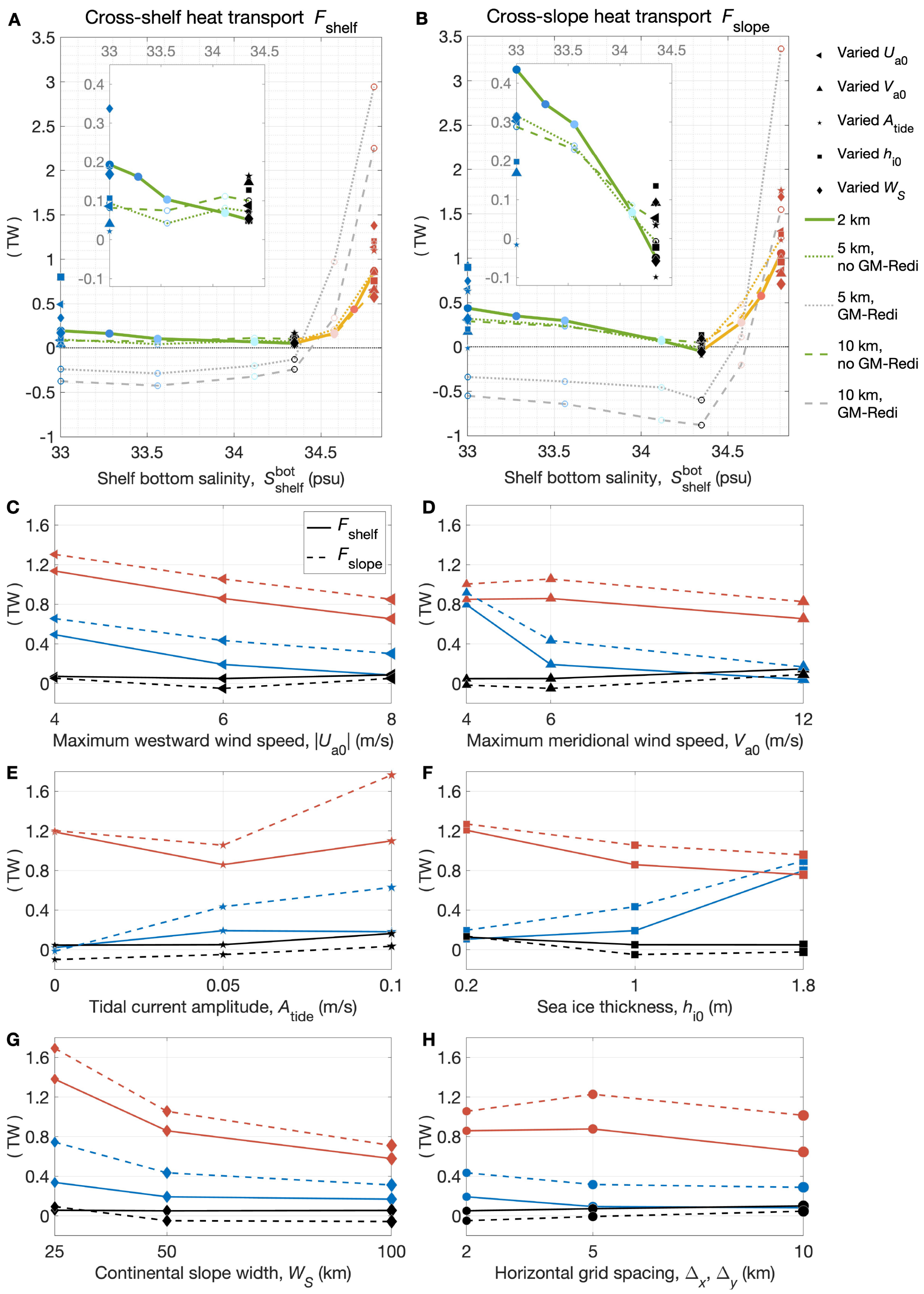
A
Cross-shelf heat transport F_{shelf}
B
Cross-slope heat transport F_{slope}
Varied U_{a0}
Varied V_{a0}
Varied A_{tide}
Varied h_{i0}
Varied W_S
2 km
5 km, no GM-Redi
5 km, GM-Redi
10 km, no GM-Redi
10 km, GM-Redi
(TW)
Shelf bottom salinity, S_{shelf}^{bot} (psu)
C
F_{shelf}
F_{slope}
Maximum westward wind speed, $|U_{a0}|$ (m/s)
D
Maximum meridional wind speed, V_{a0} (m/s)
E
Tidal current amplitude, A_{tide} (m/s)
F
Sea ice thickness, h_{i0} (m)
G
Continental slope width, W_S (km)
H
Horizontal grid spacing, Δ_x, Δ_y (km)

Fig. S2. Sensitivity of heat transferred onto the continental shelf (F_{shelf}) and to the upper part of the continental slope (F_{slope}). (**A**) vertically and zonally integrated meridional advective heat flux averaged over the shelf region (y = 50 km–75 km), in unit TW (1 TW= 10^{12} W), as a function of shelf bottom salinity. The solid, dotted, and dashed lines denote simulations with 2-km, 5-km, and 10-km resolution, respectively. The colored (green for the fresh-shelf regime and orange for the dense-shelf regime) and gray lines denote simulations with no GM-Redi eddy parameterization, and with GM-Redi, respectively. The inset plot is a zoom in of F_{shelf} in the fresh-shelf regime. For the fresh-shelf, reference, and dense-shelf cases, the sensitivity of F_{shelf} to other model parameters is indicated by markers with various shapes, with larger marker sizes indicating larger values of the corresponding parameters. (**B**) Similar to panel (**A**) but for the upper part of the continental slope (y = 125 km–150 km). (**C-H**) Sensitivity of F_{shelf} (solid lines) and F_{slope} (dashed lines) to model parameters. Blue, black, and red represent the fresh-shelf, reference, and dense-shelf cases, respectively. The simulations with 5-km or 10-km resolution in panel (**H**) are run without the GM-Redi eddy parameterization.

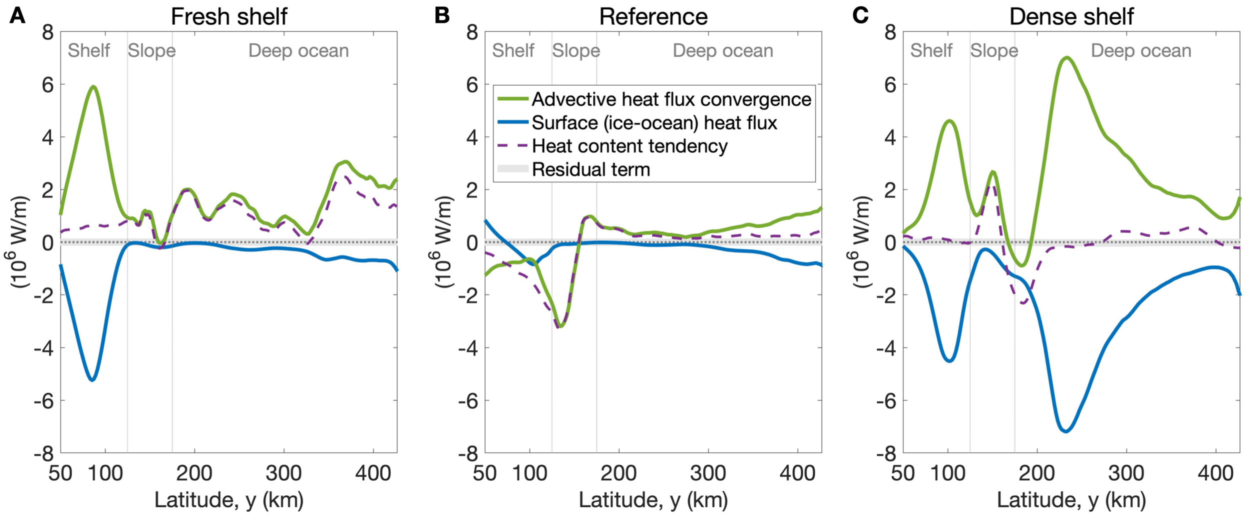

Fig. S3. Zonally and vertically integrated heat budget for simulations with 2-km horizontal resolution. (**A**) The fresh-shelf case. (**B**) The reference case. (**C**) The dense-shelf case. The residual term = (advective heat flux convergence) + (ice-ocean heat flux) – (heat content tendency). The heat content tendency is defined as $c_p \rho_0 \oint \left[\int_{z=\eta_b}^{z=0} (\partial T/\partial t) dz\right] dx$, where c_p is the specific heat capacity, ρ_0 is the reference density, η_b is the seafloor elevation, and T is the potential temperature. Positive values are associated with an increase in ocean temperature. Note that for the fresh- and dense-shelf cases, the advective heat flux convergence is balanced by ocean-to-sea ice heat flux over the continental shelf.

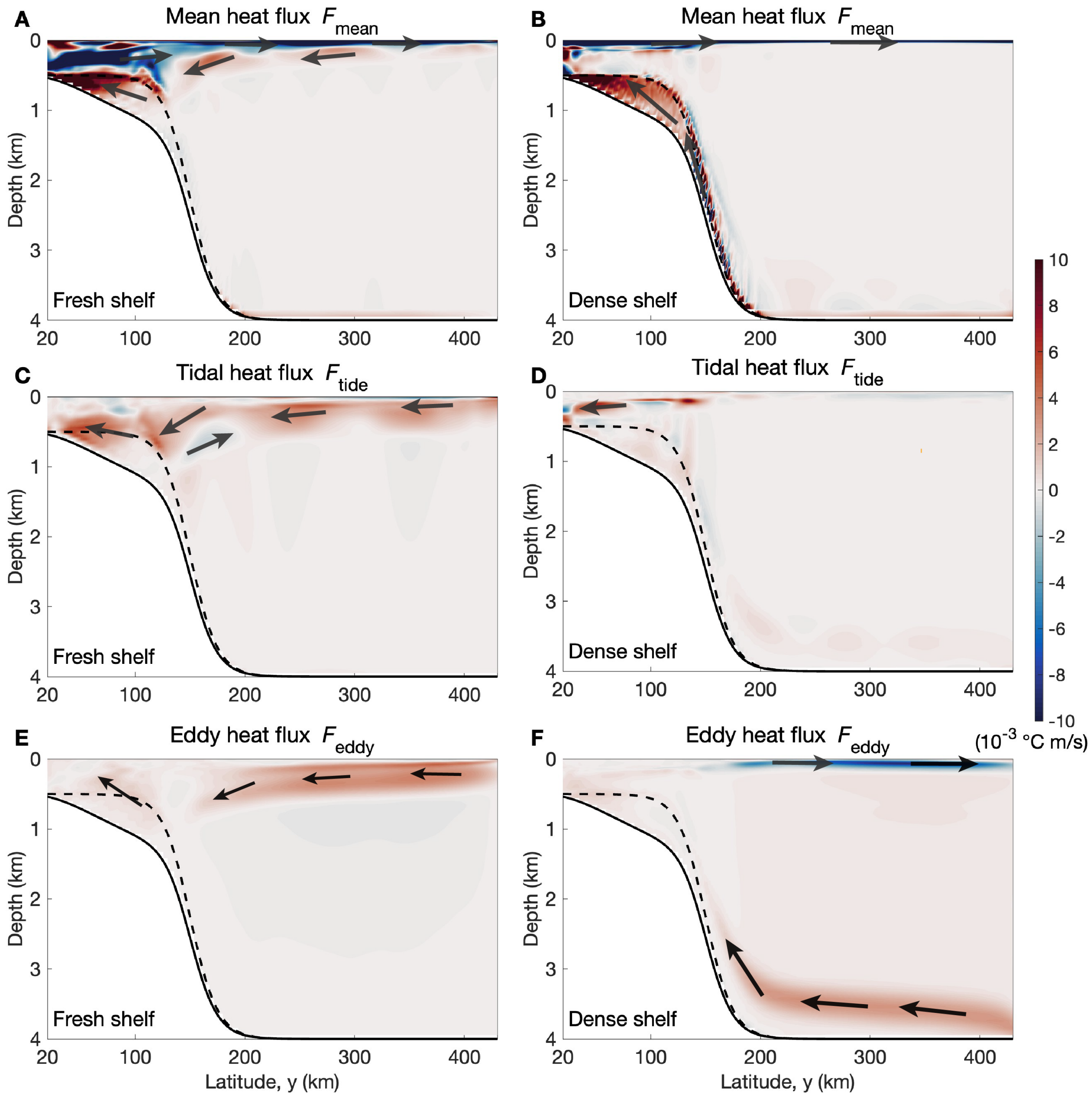

Fig. S4. Temporal decomposition of the zonal-mean southward advective heat flux for simulations with 2-km horizontal resolution. Mean, tidal, and eddy heat fluxes of the fresh-shelf (**A**, **C**, **E**) and dense-shelf (**B**, **D**, **F**) cases. The black solid and dashed curves denote the deepest and shallowest bathymetry at each latitude, respectively. The 20-km sponge layers at the northern and southern boundaries are not shown.

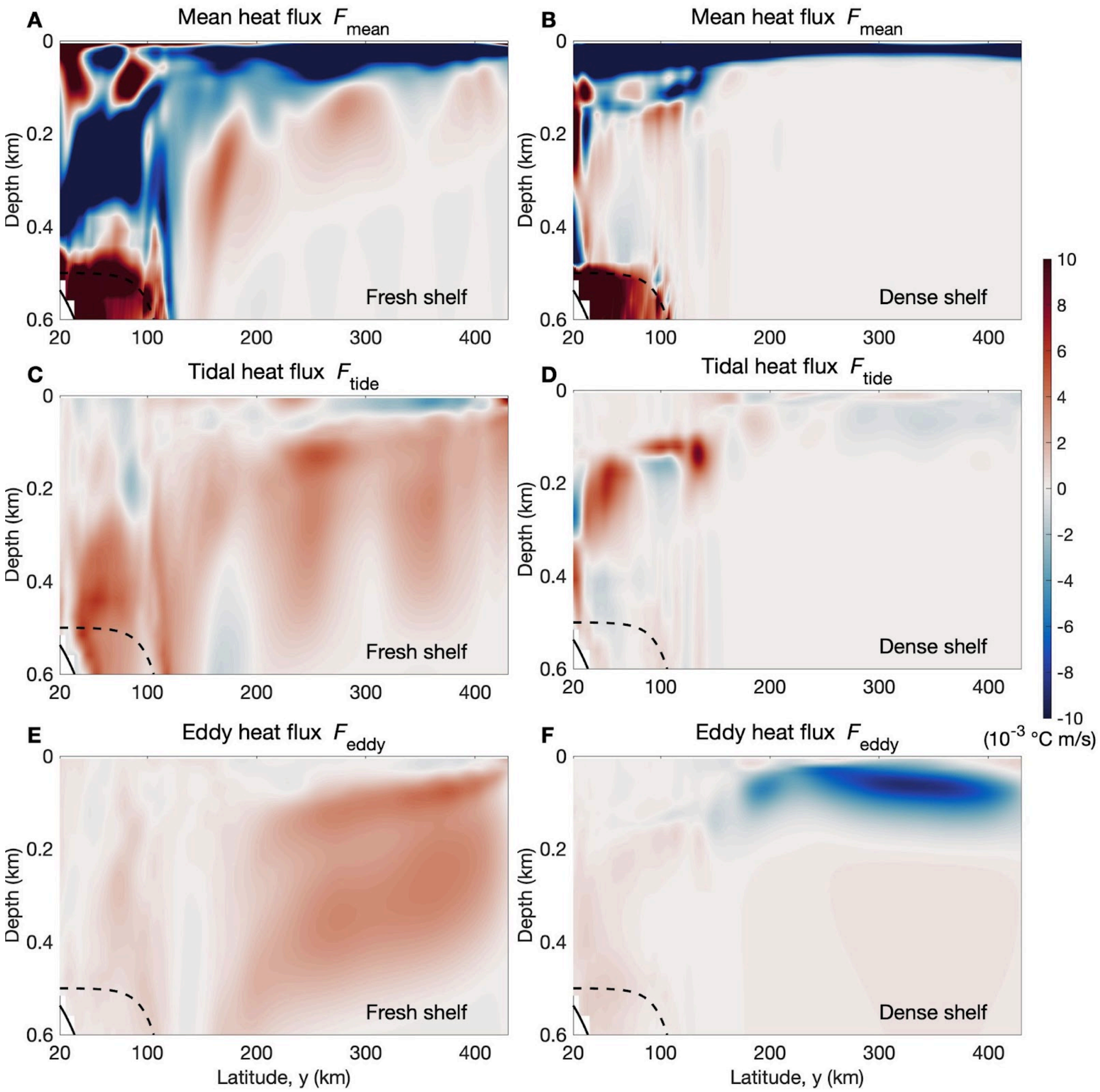

Fig. S5: Same as Fig. S4, but for the surface 600m of the ocean.

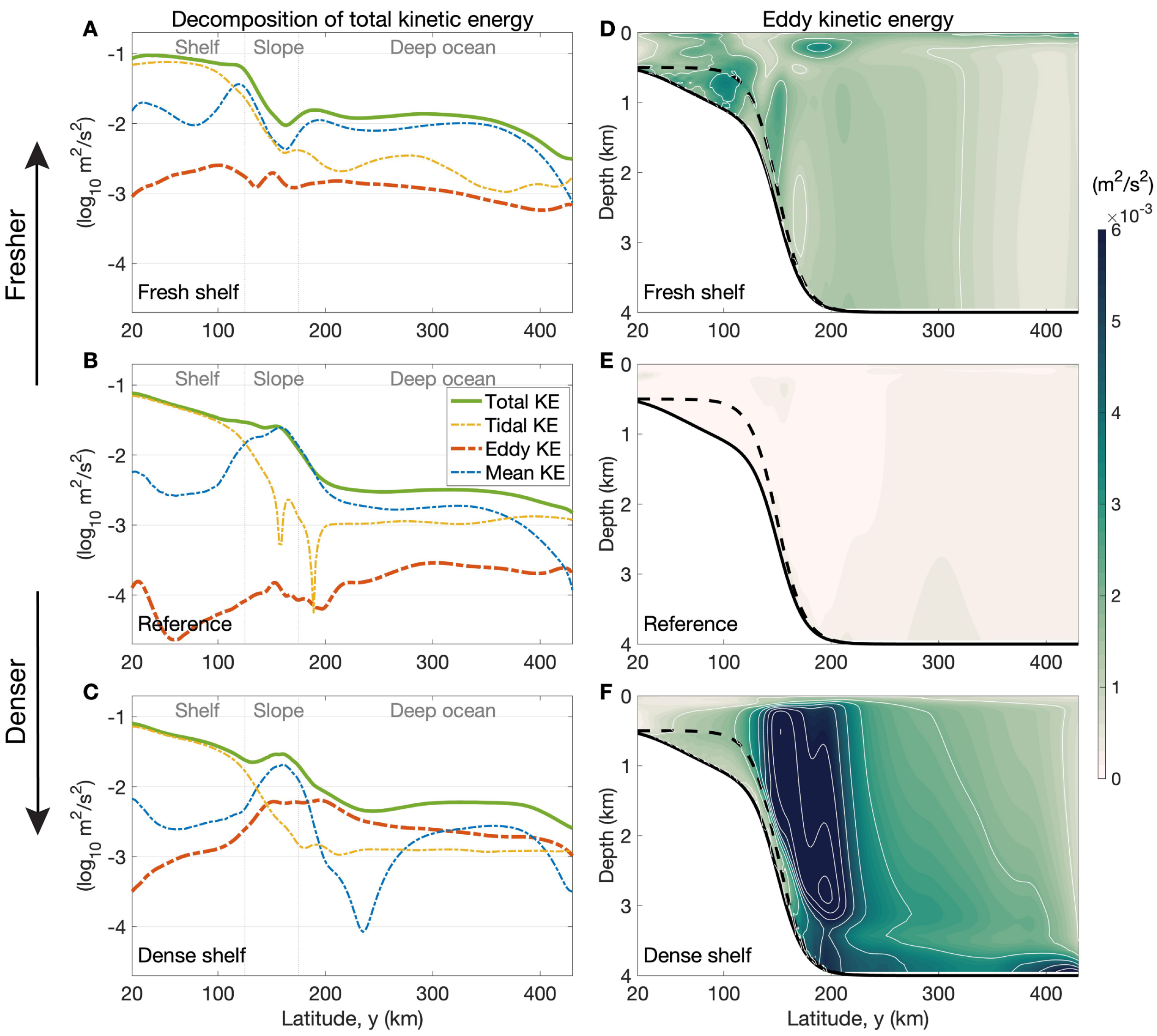

Fig. S6. Temporal decomposition of the total kinetic energy for simulations with 2-km horizontal resolution. (**A-C**) Time-mean total kinetic energy, and its tidal, eddy, and mean components in the fresh-shelf, reference, and dense-shelf cases, averaged over the zonal/vertical plane. (**D-F**) Time- and zonal-mean eddy kinetic energy in the three cases. The colored and solid white contours show intervals of 1.5×10^{-4} m^2 s^{-2} and 10^{-3} m^2 s^{-2}, respectively. The black solid and dashed curves denote the deepest and shallowest bathymetry at each latitude, respectively. The 20-km sponge layers at the northern and southern boundaries are not shown. Note the total kinetic energy and mean kinetic energy remain the same order of magnitude across simulations, while the eddy kinetic energy increases by 1~2 orders of magnitude in the fresh- and dense-shelf cases.

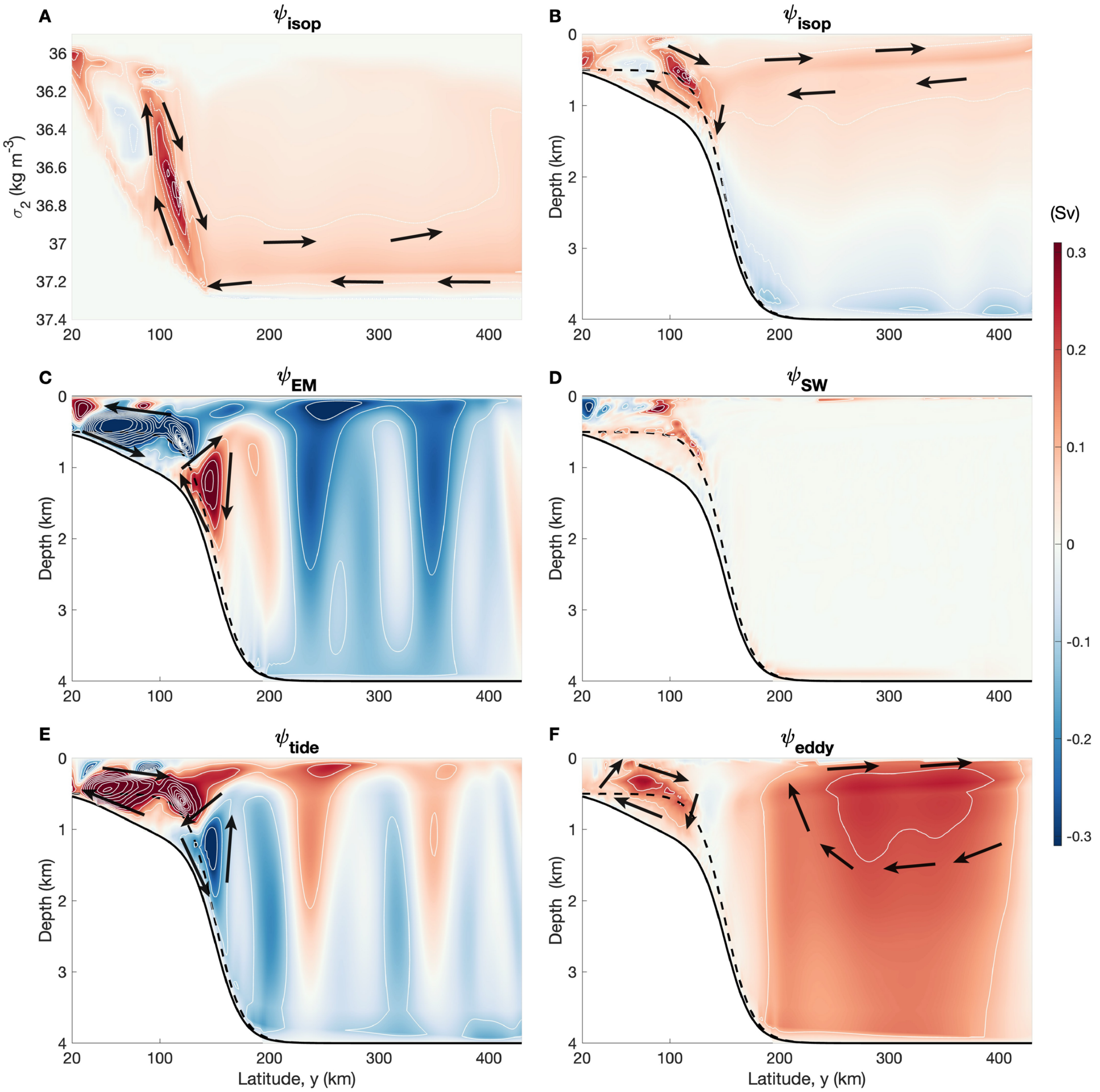

Fig. S7. Temporal decomposition of the isopycnal overturning streamfunction ($\psi_{\mathrm{isop}} = \psi_{\mathrm{EM}} + \psi_{\mathrm{SW}} + \psi_{\mathrm{eddy}} + \psi_{\mathrm{tide}}$) for the fresh-shelf simulation with 2-km horizontal resolution. Isopycnal overturning streamfunction (ψ_{isop}) with a reference depth of 2 km in potential density (σ_2) space (**A**) and z space (**B**). (**C**) Eulerian-mean overturning streamfunction (ψ_{EM}). (**D**) Standing-wave overturning streamfunction (ψ_{SW}). (**E**) Tidal overturning streamfunction (ψ_{tide}). (**F**) Transient-eddy overturning streamfunction (ψ_{eddy}). The white dashed and solid contours show intervals of 0.05 Sv (1 Sv = 10^6 m^3 s^{-1}) and 0.1 Sv, respectively. The black arrows show the direction of the overturning circulation, with positive values (red) corresponding to clockwise circulation, and negative values (blue) corresponding to counter-clockwise circulation. The black solid and dashed curves denote the deepest and shallowest bathymetry at each latitude, respectively. The 20-km sponge layers at the northern and southern boundaries are not shown. Note that the tidal component is largely compensated by the Eulerian-mean component.

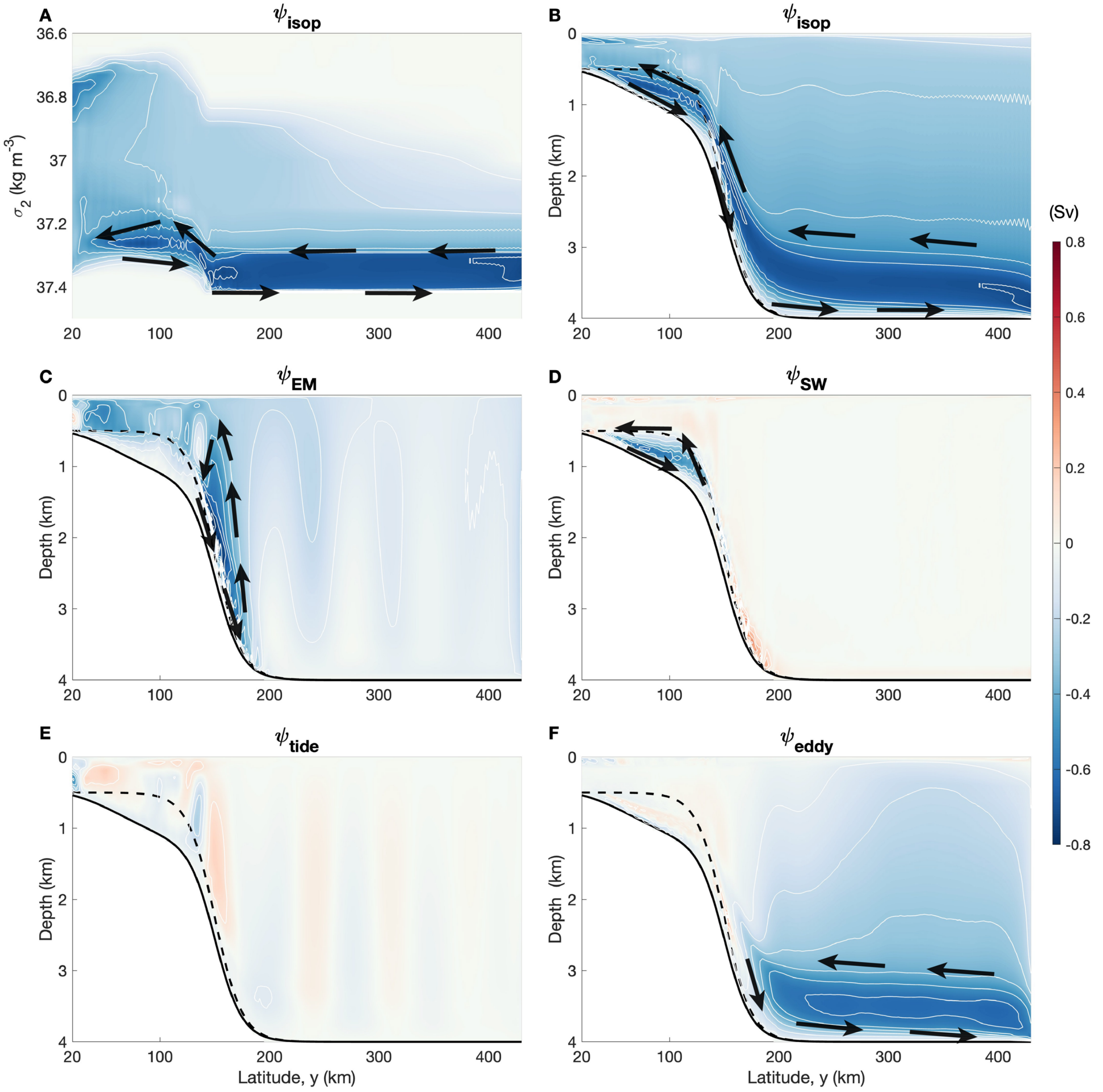

Fig. S8. Temporal decomposition of the isopycnal overturning streamfunction for the dense-shelf simulation with 2-km horizontal resolution. As Fig. S6, but for the dense-shelf case.

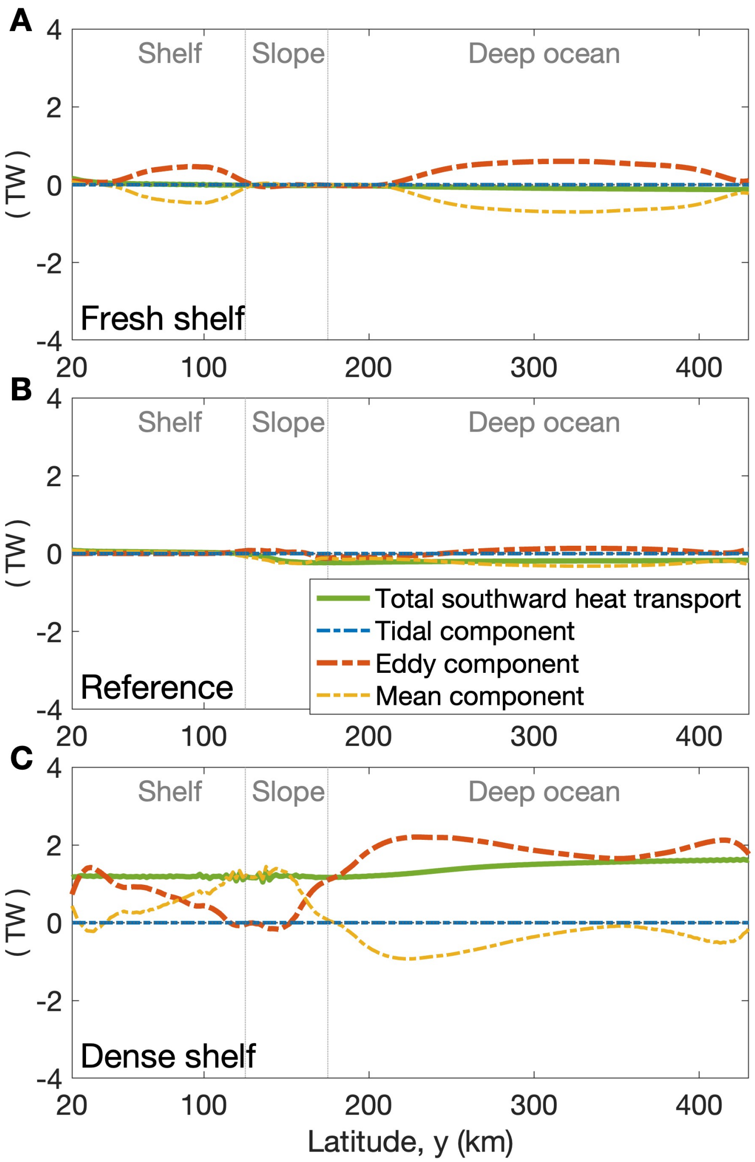

Fig. S9. Eddy/mean heat decomposition for simulations with no tides and 2-km horizontal resolution.

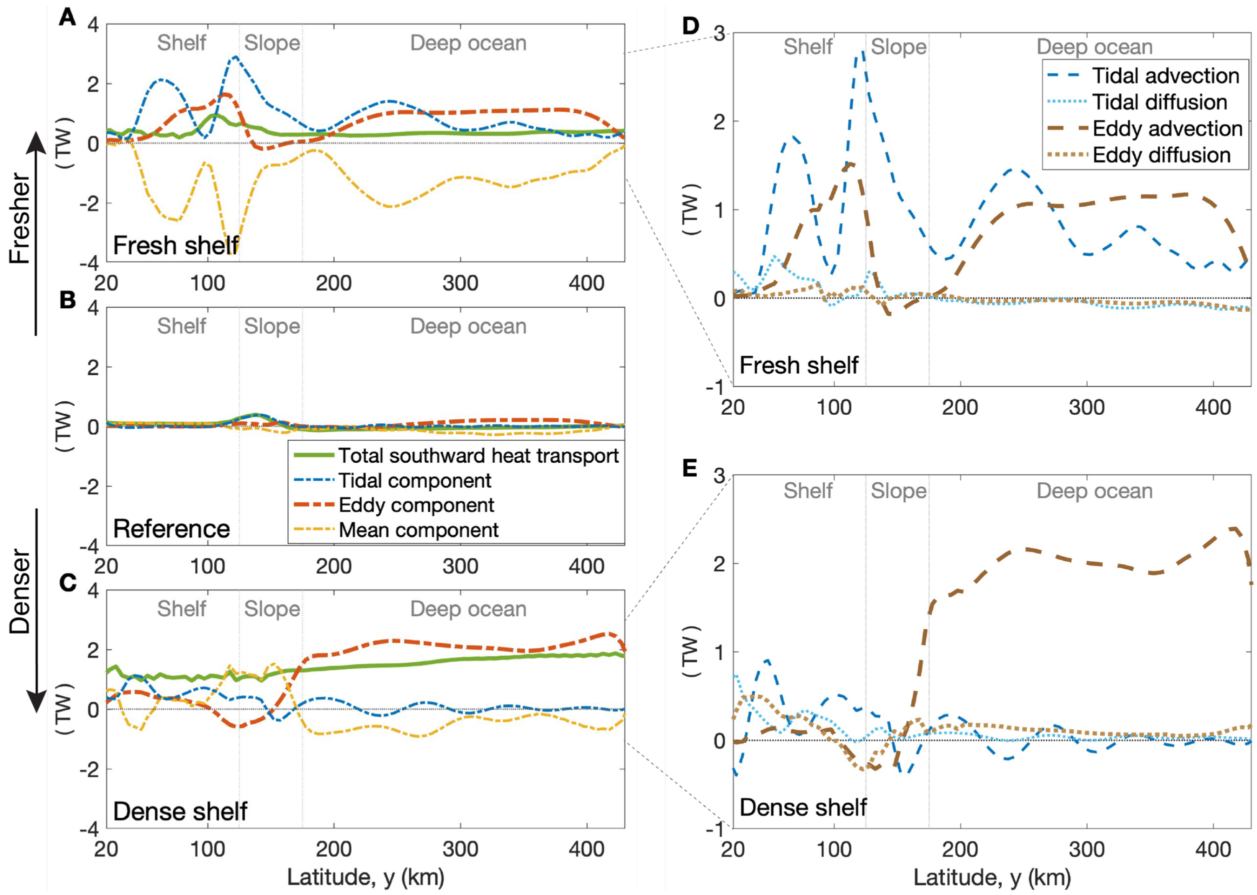

Fig. S10. Temporal decomposition of the total shoreward heat transport. As Fig. 4, but for simulations with 5-km horizontal resolution.

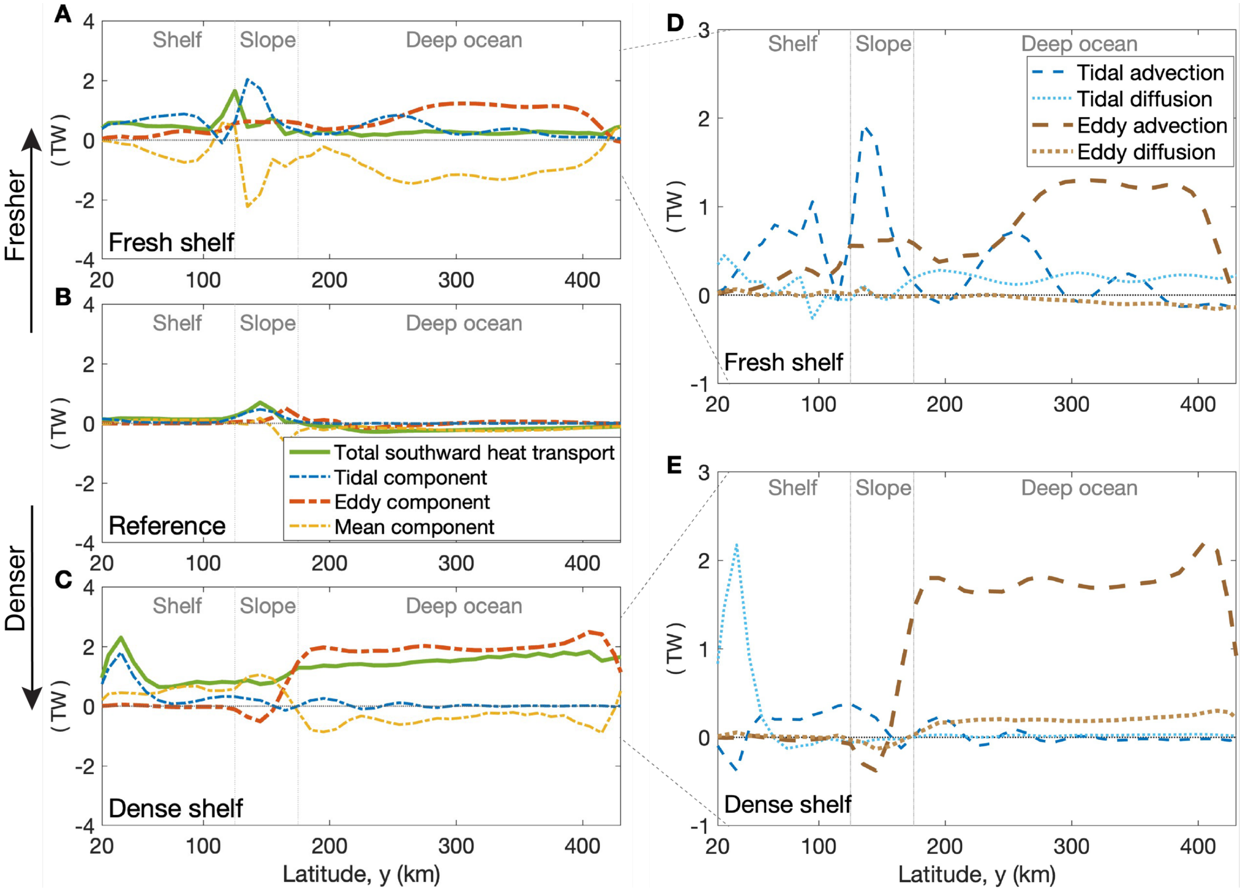

Fig. S11. Temporal decomposition of the total shoreward heat transport. As Fig. 4, but for simulations with 10-km horizontal resolution.

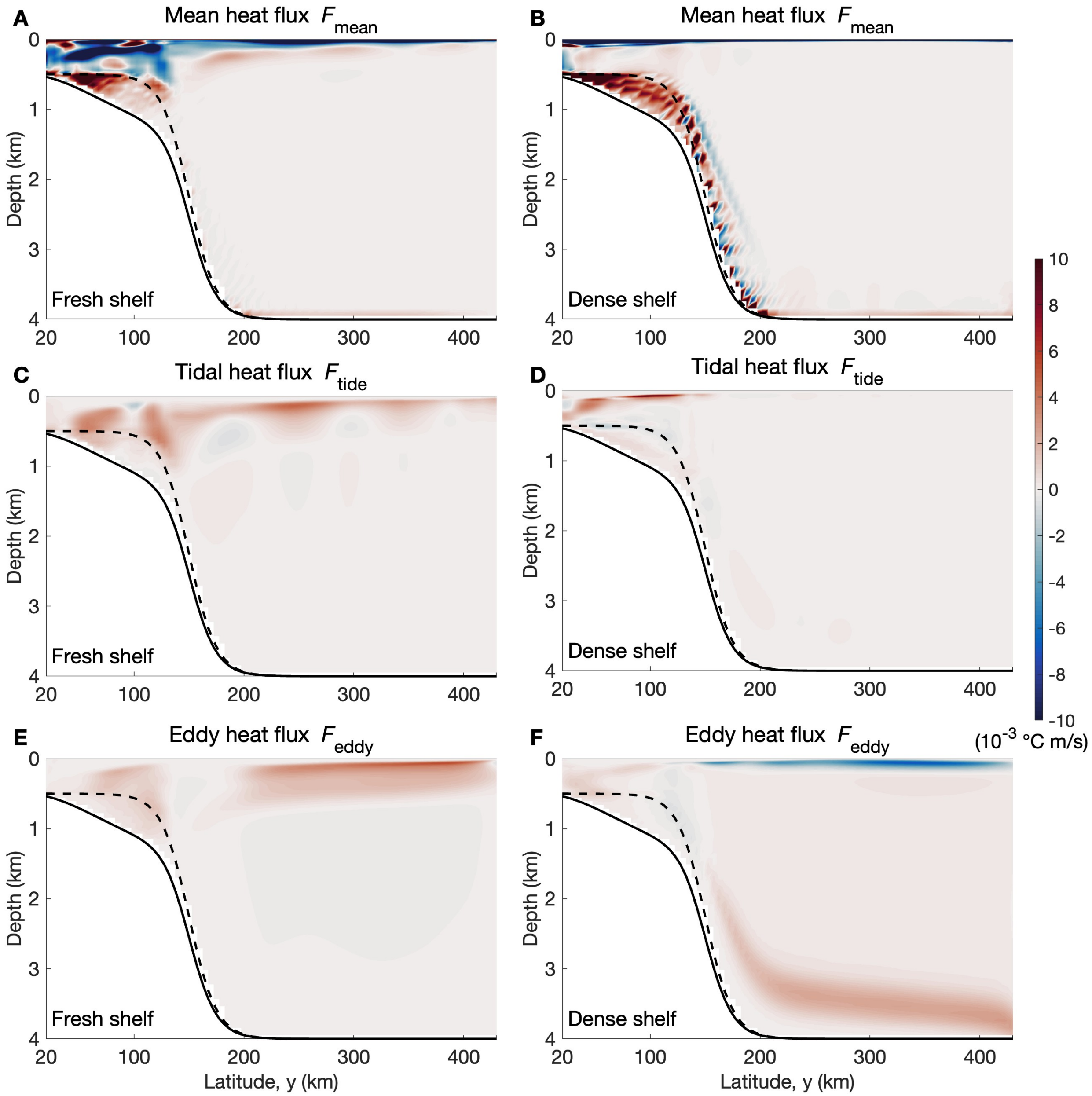

Fig. S12. Temporal decomposition of the zonal-mean southward advective heat flux. As Fig. S4, but for simulations with 5-km horizontal resolution.

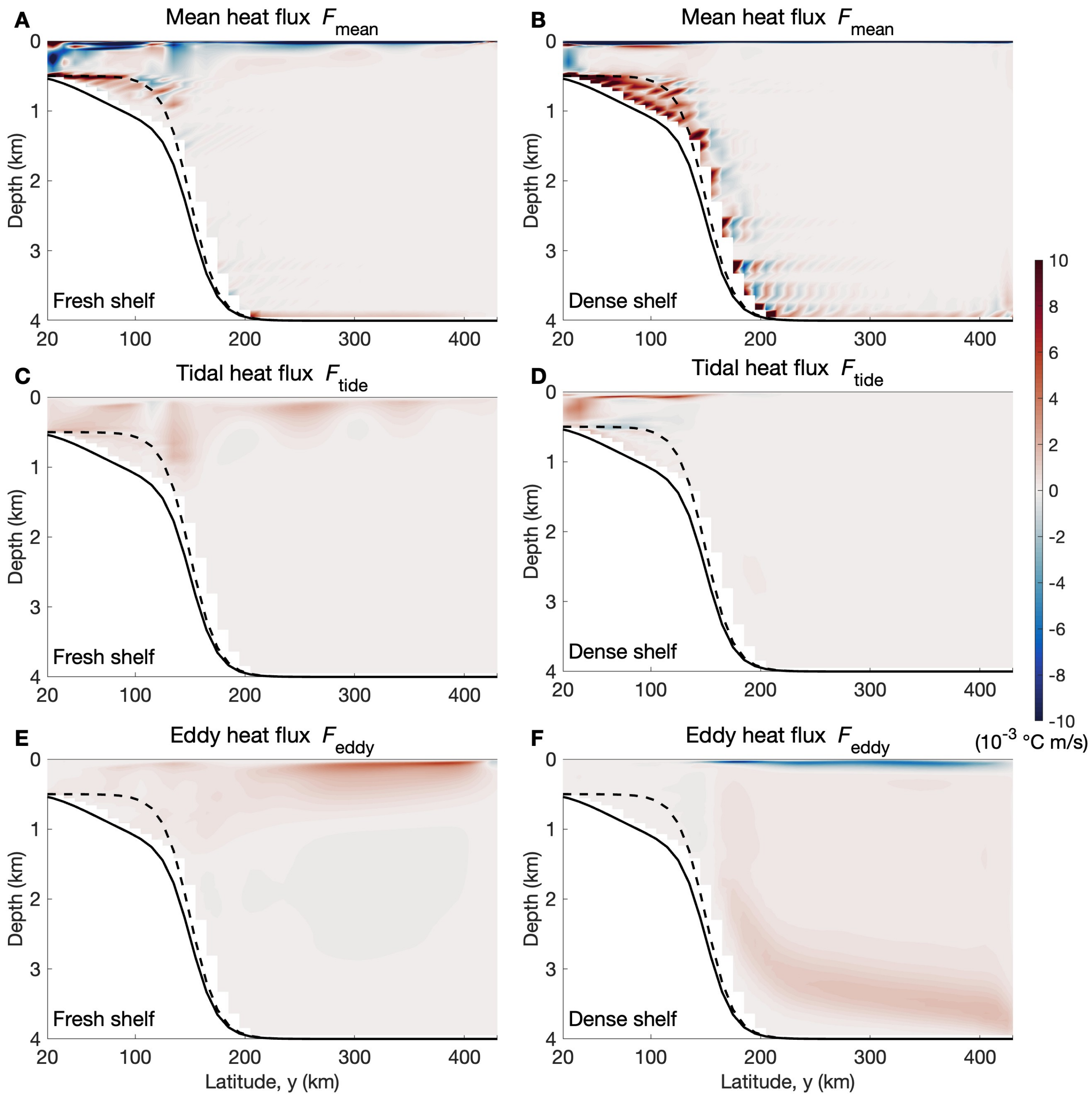

Fig. S13. Temporal decomposition of the zonal-mean southward advective heat flux. As Fig. S4, but for simulations with 10-km horizontal resolution.

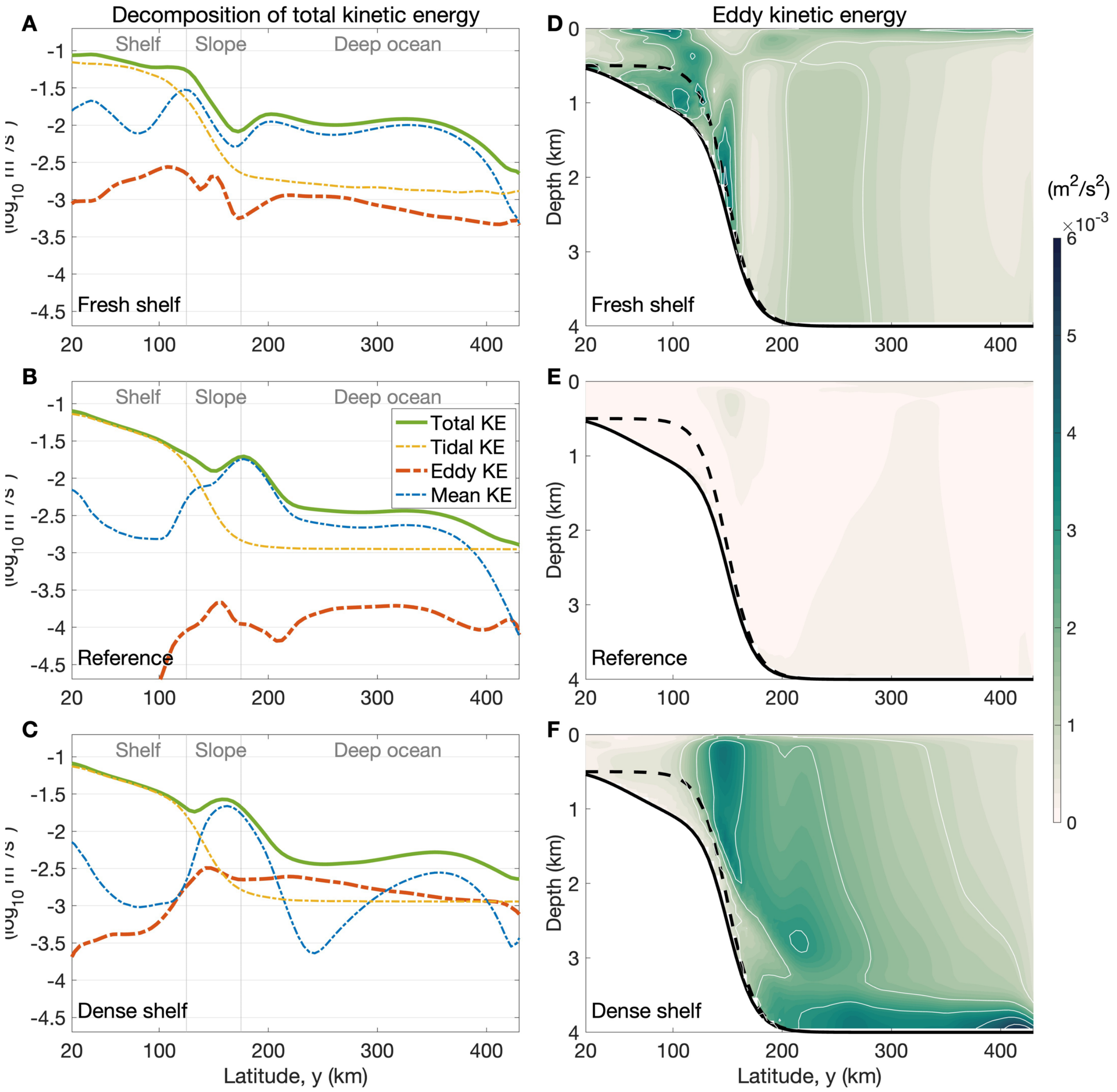

Fig. S14. Temporal decomposition of the total kinetic energy. As Fig. S6, but for simulations with 5-km horizontal resolution.

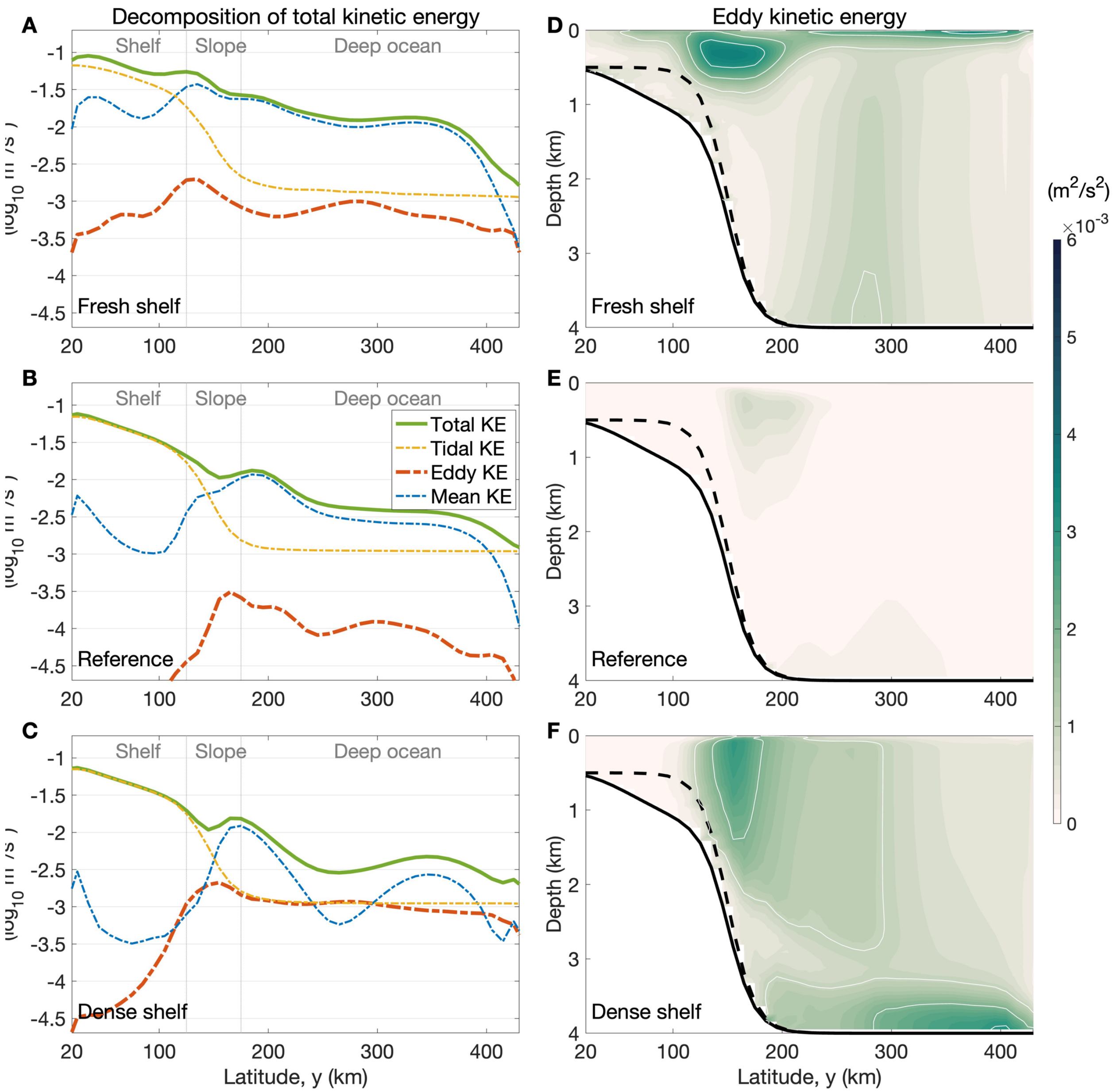

Fig. S15. Temporal decomposition of the total kinetic energy. As Fig. S6, but for simulations with 10-km horizontal resolution.

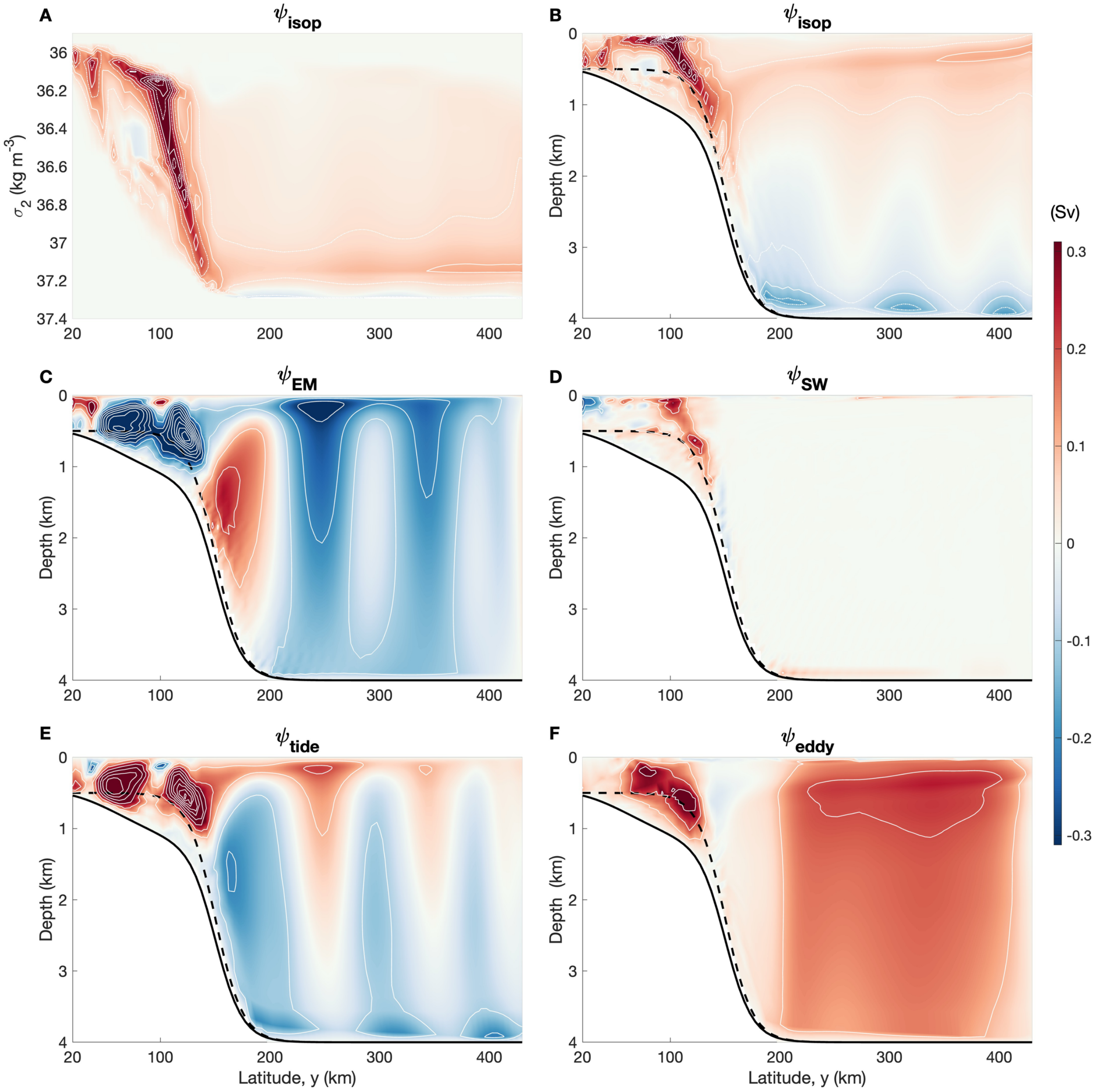

Fig. S16. Temporal decomposition of overturning streamfunction for the fresh-shelf case. As Fig. S7, but for simulations with 5-km horizontal resolution.

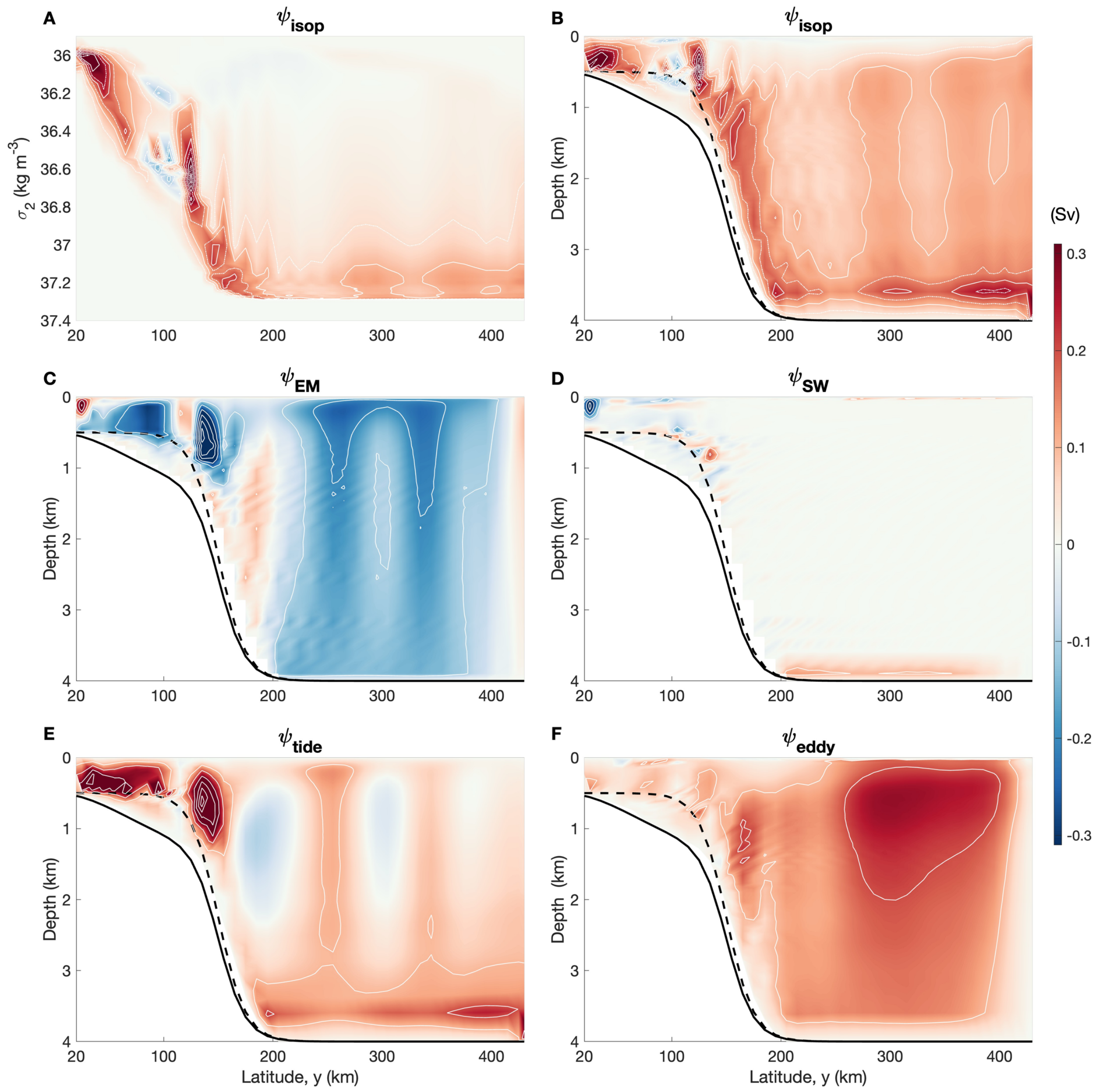

Fig. S17. Temporal decomposition of overturning streamfunction for the fresh-shelf case. As Fig. S7, but for simulations with 10-km horizontal resolution.

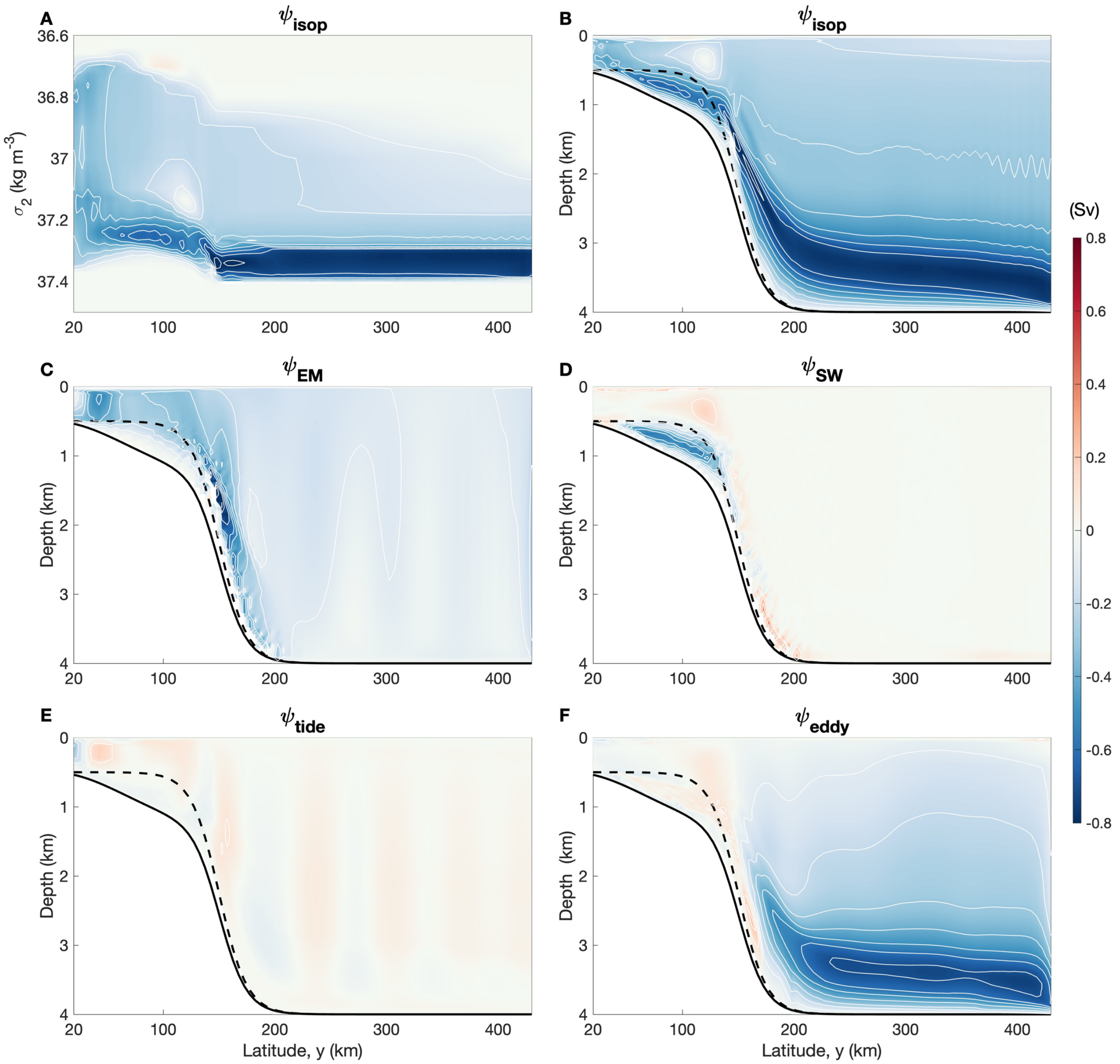

Fig. S18. Temporal decomposition of overturning streamfunction for the dense-shelf case. As Fig. S8, but for simulations with 5-km horizontal resolution.

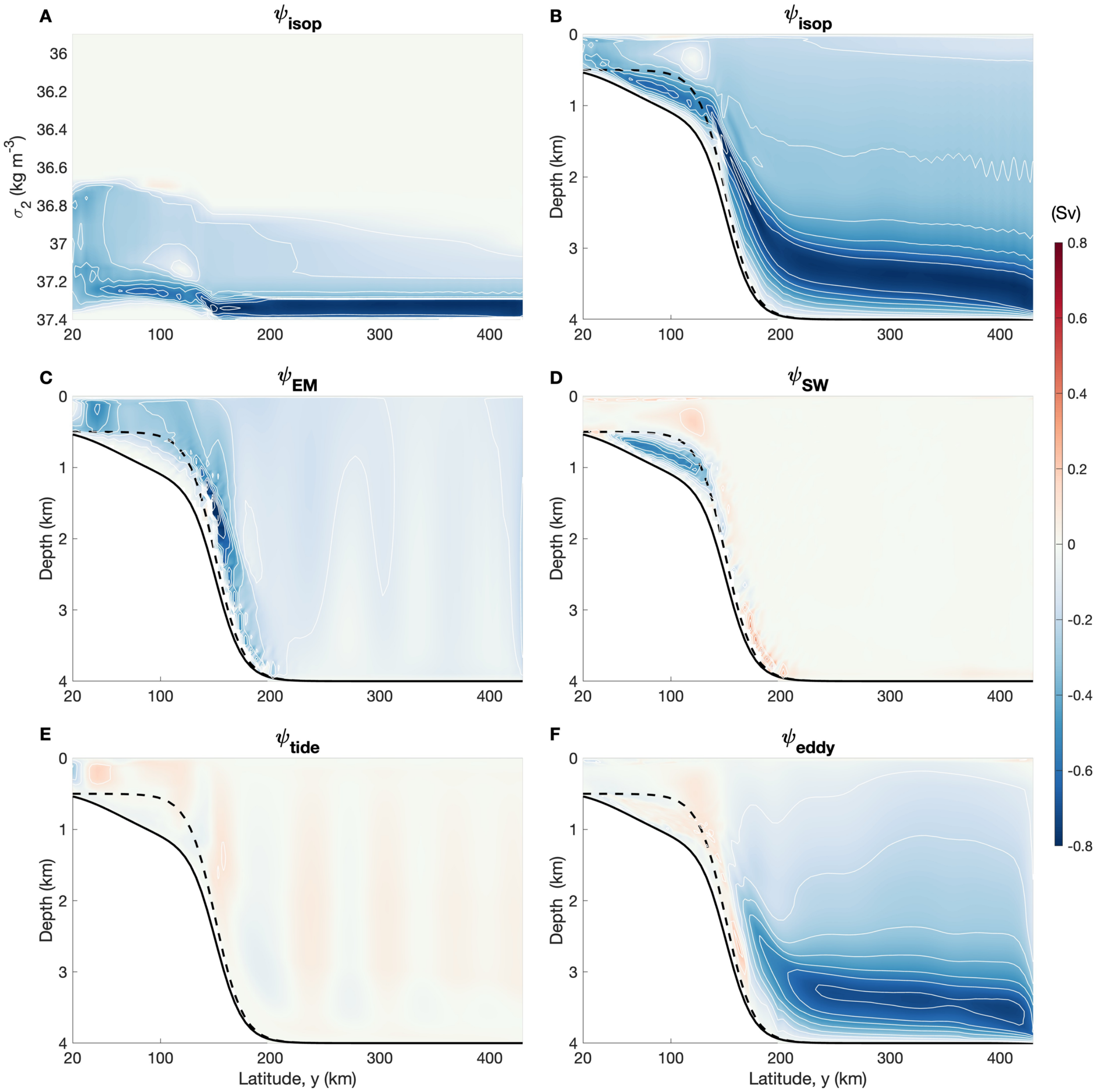

Fig. S19. Temporal decomposition of overturning streamfunction for the dense-shelf case. As Fig. S8, but for simulations with 10-km horizontal resolution.

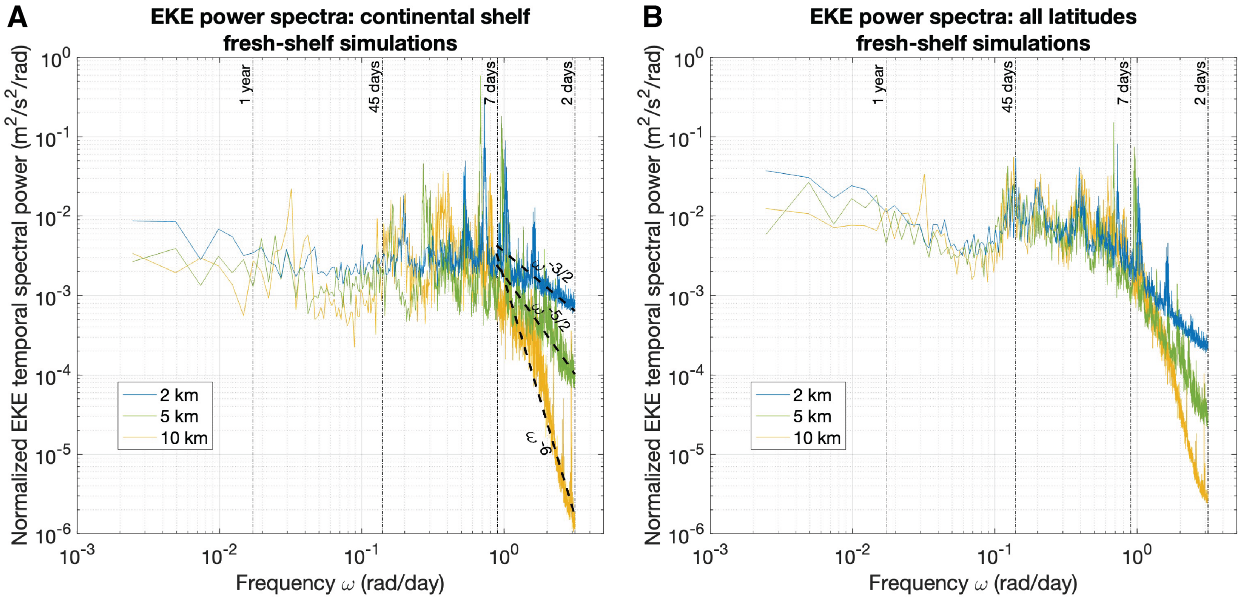

Fig. S20: EKE power spectra for fresh-shelf simulations with different horizontal resolutions, normalized by the length of the timeseries. (A) EKE spectrum over the continental shelves (latitude y=20–125 km and depth z=0–500 m). (B) Surface 500 m EKE spectrum for all latitudes (latitude y=20–430 km excluding the 20km sponge layers at the southern and northern boundaries, and depth z=0–500 m).

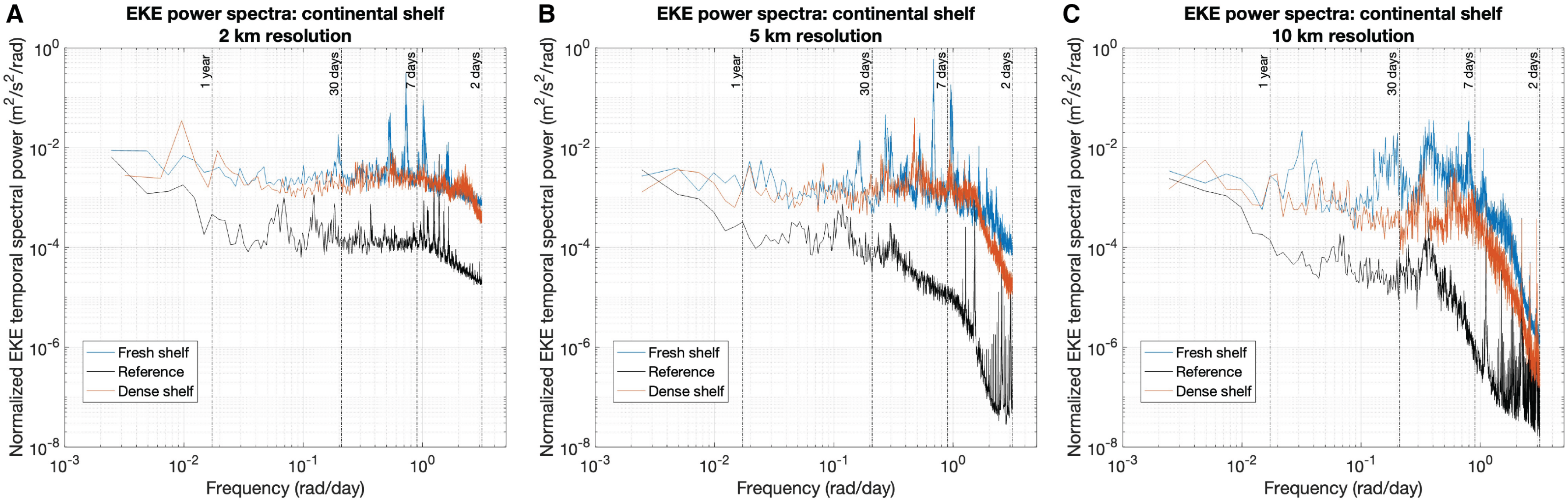

Fig. S21: EKE power spectra over the continental shelves (latitude y=20–125 km, depth z=0–500 m), normalized by the length of the timeseries. (A) Fresh-shelf, reference, and dense-shelf simulations in the 2-km model. (B) 5-km model. (C) 10-km model.

Δ_x, Δ_y (km)	$S_{\mathrm{south}}^{\mathrm{surf}}$ (psu)	$S_{\mathrm{south}}^{\mathrm{bot}}$ (psu)	U_{a0} (m/s)	V_{a0} (m/s)	A_{tide} (m/s)	h_{i0} (m)	W_S (km)
**2**, 5, 10	**33**	**33**	**-6**	**6**	**0.05**	**1.0**	**50**
2	33.28	33.28	-6	6	0.05	1.0	50
**2**, 5, 10	33.56	33.56	-6	6	0.05	1.0	50
**2**, 5, 10	34.12	34.12	-6	6	0.05	1.0	50
**2**, 5, 10	**34.12**	**34.12+Δ*S***	**-6**	**6**	**0.05**	**1.0**	**50**
**2**, 5, 10	34.12	34.12+2Δ*S*	-6	6	0.05	1.0	50
2	34.12	34.12+2.5Δ*S*	-6	6	0.05	1.0	50
**2**, 5, 10	**34.12**	**34.12+3Δ*S***	**-6**	**6**	**0.05**	**1.0**	**50**
2	33, 34.12	33, 34.12+Δ*S*, 34.12+3Δ*S*	**-4**	6	0.05	1.0	50
2	33, 34.12	33, 34.12+Δ*S*, 34.12+3Δ*S*	**-8**	6	0.05	1.0	50
2	33, 34.12	33, 34.12+Δ*S*, 34.12+3Δ*S*	-6	**4**	0.05	1.0	50
2	33, 34.12	33, 34.12+Δ*S*, 34.12+3Δ*S*	-6	**12**	0.05	1.0	50
2	33, 34.12	33, 34.12+Δ*S*, 34.12+3Δ*S*	-6	6	**0.00**	1.0	50
2	33, 34.12	33, 34.12+Δ*S*, 34.12+3Δ*S*	-6	6	**0.10**	1.0	50
2	33, 34.12	33, 34.12+Δ*S*, 34.12+3Δ*S*	-6	6	0.05	**0.2**	50
2	33, 34.12	33, 34.12+Δ*S*, 34.12+3Δ*S*	-6	6	0.05	**1.8**	50
2	33, 34.12	33, 34.12+Δ*S*, 34.12+3Δ*S*	-6	6	0.05	1.0	**25**
2	33, 34.12	33, 34.12+Δ*S*, 34.12+3Δ*S*	-6	6	0.05	1.0	**100**

Table S1. List of experiments. Δ_x and Δ_y are the horizontal grid spacings in the zonal and meridional direction, respectively. $\Delta S = 0.23$ psu is the vertical difference in the restoring salinity at the southern boundary between the sea surface ($S_{\mathrm{south}}^{\mathrm{surf}}$) and the seafloor of the continental shelf (500 m depth, $S_{\mathrm{south}}^{\mathrm{bot}}$) of the reference case. U_{a0} and V_{a0} are the zonal (along-slope, positive eastward) and meridional (cross-slope, positive northward) wind speed at the southern boundary, respectively. The maximum meridional wind speed is selected to be larger than the maximum zonal wind speed to keep the zonal wind stress ($\tau_a^x = \rho_a C_{ai}\sqrt{u_a^2 + v_a^2}\, u_a$, where ρ_a is the air density, C_{ai} is the air-ice drag coefficient, and u_a and v_a are the zonal and meridional wind speeds, respectively) comparable for the simulations with U_{a0} = - 8m/s and V_{a0} = 12 m/s. A_{tide} is the prescribed barotropic tidal current amplitude at the northern boundary. h_{i0} is the restoring sea ice thickness at the southern boundary, which is also the initial sea ice thickness across the domain. W_S is the continental slope width. The boldface shows the three experiments mainly described in this article (the fresh-shelf, reference, and dense-shelf cases), as well as perturbation simulations.
